# Research Progress of PROTACs in Breast Cancer: Subtype-Oriented Target Landscape, Clinical Stratification Evidence, and Engineering Strategies for Translation

**DOI:** 10.3390/biomedicines14040835

**Published:** 2026-04-06

**Authors:** Senyang Guo, Jianhua Liu, Hongmei Zheng, Xinhong Wu

**Affiliations:** Breast Cancer Center, Hubei Cancer Hospital, Tongji Medical College, Huazhong University of Science and Technology, National Key Clinical Specialty Construction Discipline, Hubei Provincial Clinical Research Center for Breast Cancer, Wuhan Clinical Research Center for Breast Cancer, No. 116 ZhuoDaoquan South Road, Wuhan 430079, China; m13598664134@163.com (S.G.); 2019183020064@whu.edu.cn (J.L.)

**Keywords:** proteolysis-targeting chimera (PROTAC), targeted protein degradation, breast neoplasms, estrogen receptor α (ERα) mutations, cereblon (CRBN) E3 ligase, von Hippel–Lindau (VHL) E3 ligase

## Abstract

Molecular subtype–guided therapy for breast cancer (BC) remains limited in a subset of patients by suboptimal efficacy, acquired resistance, and the presence of “undruggable” targets. Proteolysis-targeting chimeras (PROTACs) represent a targeted protein degradation (TPD) strategy that differs fundamentally from conventional occupancy-driven inhibition. By inducing ubiquitination of a protein of interest and subsequent proteasomal degradation, PROTACs can directly reduce pathogenic protein abundance and potentially abrogate non-catalytic or scaffolding functions, thereby enabling more durable pathway suppression in selected resistance contexts. This review comprehensively summarizes the mechanisms of action, key molecular design elements, and the developmental landscape of PROTACs, and maps target selection and research progress across BC molecular subtypes. In hormone receptor–positive/HER2-negative BC, clinical translation is most advanced for estrogen receptor alpha-directed PROTACs; Phase III evidence indicates biomarker-dependent efficacy, with clearer benefit signals in resistant subgroups such as estrogen receptor 1 mutations, suggesting that the net clinical benefit of TPD is more likely to be realized through precision stratification. In contrast, in solid-tumor settings, including human epidermal growth factor receptor 2 (HER2)-positive BC and triple-negative breast cancer, PROTAC translation is more frequently constrained by an “exposure–selectivity–therapeutic window” trade-off driven by physicochemical liabilities, insufficient tumor penetration, and broad target expression. Accordingly, engineering strategies—such as antibody/aptamer-mediated targeted delivery, stimulus-responsive prodrugs, nanocarriers, and local administration—are emerging as decisive approaches to enable safe and effective clinical implementation. Looking forward, further progress of PROTACs in BC will depend on expanding the spectrum of E3 ubiquitin ligases and recruitment modalities, establishing predictable and dynamically monitorable biomarker systems, optimizing rational combination/sequencing regimens with exposure- and schedule-guided dosing, and advancing scalable manufacturing and quality control capabilities, thereby translating mechanistic advantages of TPD into verifiable precision-therapy applications.

## 1. Introduction

Breast cancer (BC) is one of the most common malignancies in women worldwide and represents a major public health burden [1]. With the evolution of molecular subtyping, subtype-guided precision therapy has improved outcomes for selected patient populations: hormone receptor-positive/HER2-negative BC (approximately 70% of cases) relies on endocrine therapy as the therapeutic backbone; human epidermal growth factor receptor 2 (HER2)-positive BC benefits from anti-HER2-targeted agents and antibody–drug conjugates (ADCs); and triple-negative breast cancer (TNBC), which lacks actionable receptor targets, is still largely treated with chemotherapy in the systemic setting [2,3,4,5]. However, in advanced/metastatic BC, further improvements in overall survival (OS) have plateaued, and acquired resistance remains pervasive [6,7]. Treatment in this setting is often limited by tumor heterogeneity (across primary and metastatic lesions and within individual tumors), as well as by components of the tumor microenvironment (TME)—for example, stromal and vascular barriers that constrain drug penetration and dynamic immune-stromal-neural crosstalk that can promote adaptive resistance [7]. Importantly, such neural–microenvironmental remodeling may also indirectly shape vascular function, extracellular-matrix organization, and immune infiltration, thereby influencing intratumoral drug distribution and reinforcing exposure-limited resistance—an issue that is particularly relevant for beyond-Rule-of-Five modalities such as proteolysis-targeting chimeras (PROTACs) [7]. In parallel, advanced patient-derived models such as human organoids are increasingly being used to capture inter- and intratumoral heterogeneity, evaluate drug response, and provide a translational framework for prioritizing emerging therapeutic modalities in BC [8]. Moreover, several critical oncogenic drivers are difficult to address with conventional drug modalities because they lack druggable pockets or function primarily through protein–protein interactions and non-catalytic scaffolding activities [9]. These limitations collectively point toward novel intervention strategies, exemplified by targeted protein degradation (TPD).

Resistance mechanisms across BC subtypes collectively require more precise treatment algorithms and rational combination strategies. In estrogen receptor (ER)-positive BC, approximately 30% of patients with early-stage disease and most patients with advanced disease eventually experience progression [10,11]. Acquired mutations in the ligand-binding domain (LBD) of estrogen receptor 1 (ESR1)—such as Y537S and D538G—can confer ligand-independent activation of ERα, thereby reducing the efficacy of aromatase inhibitors (AIs) and some selective estrogen receptor degraders (SERDs) [12,13]. These alterations may further cooperate with bypass pathway activation (e.g., epidermal growth factor receptor (EGFR) amplification) to facilitate therapeutic escape [14]. In HER2-positive BC, although trastuzumab-based regimens have substantially improved survival, kinase-domain alterations, compensatory activation of downstream signaling pathways (e.g., phosphoinositide 3-kinase (PI3K)/protein kinase B (AKT)/mechanistic target of rapamycin (mTOR)), and receptor network rewiring can still drive treatment failure [15,16]. Moreover, the expanding clinical use of ADCs has introduced additional resistance layers—including antigen heterogeneity or loss, altered internalization/lysosomal trafficking, payload efflux, and adaptive pathway rewiring—which further support exploration of complementary therapeutic modalities capable of eliminating disease-driving proteins [5]. TNBC faces an even more urgent need for novel strategies due to limited targetable options and poor prognosis after resistance develops [4,17].

Conventional small-molecule inhibitors and therapeutic antibodies largely depend on sustained target occupancy to block function, and their efficacy can be constrained by the occupancy requirement, target conformational changes/mutations, target overexpression, and “undruggable” structural features [9,18]. SERDs can reduce ER protein levels and enhance pathway suppression; however, they often require high and sustained exposure and may still be compromised by ESR1 mutations, receptor resynthesis, or activation of alternative signaling programs. TPD technologies—exemplified by PROTACs—recruit an E3 ubiquitin ligase (E3) to form a ternary complex, trigger event-driven ubiquitination, and enable recyclable elimination of the protein of interest (POI). Mechanistically, this paradigm may help overcome limitations associated with “occupancy-only” inhibition or incomplete degradation in specific resistance contexts [19].

Accordingly, TPD approaches that harness endogenous cellular protein quality-control machinery—primarily the ubiquitin–proteasome system (UPS) and in some contexts the autophagy–lysosome pathway—have attracted substantial attention as strategies to eliminate pathogenic proteins [20]. Among these, PROTACs have emerged as one of the fastest-advancing modalities toward clinical translation, owing to their programmable small-molecule design and a relatively well-defined development trajectory [21].

In this review, we summarize the mechanistic basis and druggability constraints of PROTACs and, organized by BC subtype (hormone receptor-positive/HER2-negative, HER2-positive, and TNBC), map the target landscape and recent progress in the field. We highlight ER-targeting PROTACs as a representative “mechanism–medicinal chemistry–phase III biomarker-stratified evidence” continuum. We then discuss shared translational bottlenecks in solid tumors—exposure, selectivity, and therapeutic window—and synthesize engineering strategies including targeted delivery, conditional activation, cell-selective degradation, and local permeability-enhancing approaches. Finally, we outline future directions encompassing expansion of the E3 ligase repertoire and recruitment strategies, establishment of predictive and dynamically monitorable biomarkers, rational combination/sequencing with optimized dose scheduling, and scalable manufacturing and quality control to enable precision implementation of TPD in BC. A cross-subtype comparison is provided in Table 1.

## 2. PROTACs and UPS-Mediated TPD: Mechanism, Design, and Technological Evolution

### 2.1. Mechanism of Action: UPS-Driven Ternary Complex Formation, Ubiquitination, and Event-Driven Degradation

PROTACs enable a paradigm shift from “occupancy-driven” inhibition to “event-driven” degradation [24]. At the core of this modality is the hijacking of a highly conserved intracellular protein quality-control machinery, the UPS. The UPS catalyzes the covalent conjugation of ubiquitin (Ub) to substrate proteins through an enzymatic cascade involving the E1 ubiquitin-activating enzyme (E1), E2 ubiquitin-conjugating enzyme (E2), and E3. E1 activates Ub and transfers it to E2, whereas E3 confers substrate specificity and facilitates Ub transfer and polyubiquitin chain formation. Polyubiquitinated proteins are subsequently recognized and degraded by the 26S proteasome [67,68,69].

PROTACs are heterobifunctional small molecules typically composed of three modules: a POI-binding ligand, an E3 ligase ligand (E3 recruiter), and a linker connecting the two [70,71]. Upon entering cells, a PROTAC simultaneously engages the POI and the E3 ligase to induce formation of a transient POI–PROTAC–E3 ternary complex, thereby positioning the POI for Ub transfer from E2~Ub and triggering its polyubiquitination, followed by proteasomal degradation [72].

Unlike conventional inhibitors, a PROTAC can dissociate from the complex after a successful ubiquitination/degradation event and participate in additional rounds of target engagement, exhibiting a “catalytic” turnover feature; in principle, a single degrader molecule may eliminate multiple POI molecules [23]. In practice, degradation efficiency is constrained by multiple factors, including the kinetics of ternary complex assembly/disassembly, the intracellular free (effective) concentration of the degrader, and the activity of drug efflux transporters [73,74,75].

**Figure 1:**
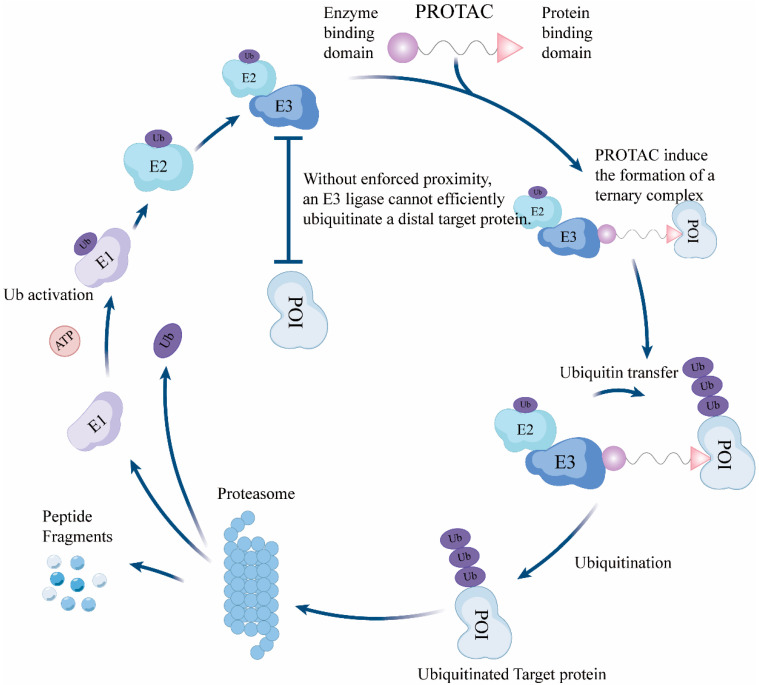
Schematic illustration of the PROTAC-mediated TPD mechanism: Induction of a ternary complex leading to ubiquitination of the POI and subsequent degradation by the proteasome.

Mechanistically, two phenomena are particularly relevant for drug discovery and optimization. First, the “hook effect” describes a bell-shaped concentration–response relationship in which degradation increases within a certain concentration window but diminishes at higher concentrations because independent saturation of the POI or E3 ligase favors non-productive binary complexes and reduces productive ternary complex formation [76,77]. Accordingly, optimization must balance binary binding affinities, ternary complex cooperativity, and the intracellular effective concentration window. Second, “ternary complex–driven selectivity” indicates that cellular selectivity is not solely determined by the binary affinities of the two ligands; rather, it is shaped by cooperativity at the ternary interface, overall conformational stability, and cell-context-dependent factors, which together can generate “emergent selectivity” beyond what would be predicted from binary binding alone [66,78,79]. Therefore, evaluation of degraders should integrate binary engagement, ternary complex cooperativity, and the effective concentration window that supports robust degradation. The PROTAC-mediated TPD mechanism is summarized in Figure 1.

### 2.2. Medicinal Chemistry Design Elements: Coordinated Optimization of the POI Ligand, E3 Recruiter, and Linker

Efficient PROTACs require coordinated optimization of three components—the POI-binding ligand, the E3 ligase ligand (E3 recruiter), and the linker—because these elements jointly determine ternary complex formation, degradation efficiency, and drug-like properties.

POI ligands are frequently derived from existing inhibitors or binders; their affinity and binding pose provide the foundation for productive degradation. However, unlike the classical inhibitor paradigm in which “higher affinity equals higher potency”, PROTACs are generally more tolerant with respect to POI-ligand affinity: with appropriate linker engineering and improved ternary complex cooperativity, ligands with only moderate affinity can still yield robust degradation activity [80].

The choice of E3 recruiter directly influences tissue/cell bias of degradation and the potential resistance landscape, and thus represents a key strategic variable. At present, the two most widely used systems are cereblon (CRBN) and von Hippel–Lindau (VHL). Ligands for the CRL4^CRBN complex (thalidomide, lenalidomide, pomalidomide, and their derivatives) emerged from mechanistic studies of immunomodulatory drugs; these agents bind the CRBN–DDB1–CUL4A complex and reprogram substrate recognition, thereby promoting the recruitment and degradation of neosubstrates such as IKZF1/3 [81,82,83]. This “reprogrammable” feature has made CRBN one of the most commonly exploited E3 ligases for PROTAC development [84]. In contrast, VHL recruiters were developed by leveraging the physiological degradation mechanism of hypoxia-inducible factor-1α (HIF-1α). By mimicking the hydroxyproline-containing recognition motif, high-affinity small molecules such as VH032 and VH298 were developed to efficiently recruit VHL [85,86]. Beyond CRBN and VHL, additional E3 ligase ligands—including those engaging the inhibitor of apoptosis protein (IAP) family (e.g., cIAP1), mouse double minute 2 homolog (MDM2), and DCAF16—are being actively explored to expand the usable E3 repertoire, reduce dependence on any single ligase pathway, and potentially mitigate resistance linked to E3 loss or rewiring [56,87,88].

The linker is not merely a spacer; it is a central determinant of potency, selectivity, and developability. Linker length, rigidity, and chemical composition collectively influence cell permeability, ternary complex conformational stability, and pharmacokinetic (PK) behavior. For a given POI–E3 pair, there is often an “optimal length window”, and deviations toward overly short or overly long linkers can markedly reduce activity [80]. In addition, the exit vector (attachment site) on each ligand is critical. For example, in early ER-targeting PROTACs, introducing the linker at the C-7α position of an estradiol scaffold produced stronger degradation than alternative substitution patterns [89]. Accordingly, systematic linker optimization is frequently the key step to simultaneously enhance cellular potency and improve drug-like properties.

In recent years, PROTAC design has also expanded beyond the assumption that the POI ligand must be an orthosteric inhibitor. Allosteric ligands and “non-functional” binders that do not occupy the canonical active site or do not directly inhibit protein function can nevertheless serve as productive warheads for PROTAC construction, offering several potential advantages: circumventing resistance driven by orthosteric-site mutations, improving paralog selectivity by exploiting allosteric-site divergence, and, in some cases, improving global physicochemical properties to enhance oral potential. For instance, a BCR–ABL PROTAC derived from the allosteric inhibitor asciminib effectively degraded multiple resistant mutants, highlighting the potential of this strategy to address mutation-driven resistance [90]. For BC-relevant targets (e.g., ER, HER2, and AKT), continued exploration of allosteric/non-functional ligand–based PROTACs may likewise provide new chemical tools to improve the therapeutic window and manage resistance. Major E3 ligase systems used in BC PROTAC research are summarized in Table 2.

### 2.3. Technological Evolution: From Peptidic Prototypes to Small-Molecule Maturation and Clinical Translation

Since the PROTAC concept was introduced in 2001, the field has broadly progressed through three stages: proof-of-concept, full small-molecule implementation, and clinical translation [73]. Early PROTACs were largely peptides or peptide–small-molecule hybrids. These constructs recruited E3 ligases through defined peptide motifs (e.g., using a phosphorylated IκBα peptide to engage SCF^β-TRCP) to degrade targets such as MetAP-2 or ERα, thereby demonstrating the feasibility of “E3 recruitment–driven targeted degradation”. However, the poor membrane permeability of peptidic elements substantially limited further development [73,91].

A major inflection point that accelerated the field was the transition to fully small-molecule PROTACs. In 2008, the first all–small-molecule PROTAC was reported (linking the MDM2 ligand Nutlin to an androgen receptor (AR) ligand), establishing that targeted degradation could be achieved without a peptide scaffold [100]. Subsequently, the discovery of potent, drug-like small-molecule E3 recruiters further strengthened the medicinal chemistry foundation: in 2010, CRBN was identified as a direct target of thalidomide [81], and high-affinity VHL ligands were developed based on structural insights into the VHL–HIF-1α interaction [85,86], enabling the design of PROTACs with markedly improved cellular activity.

**Figure 2:**
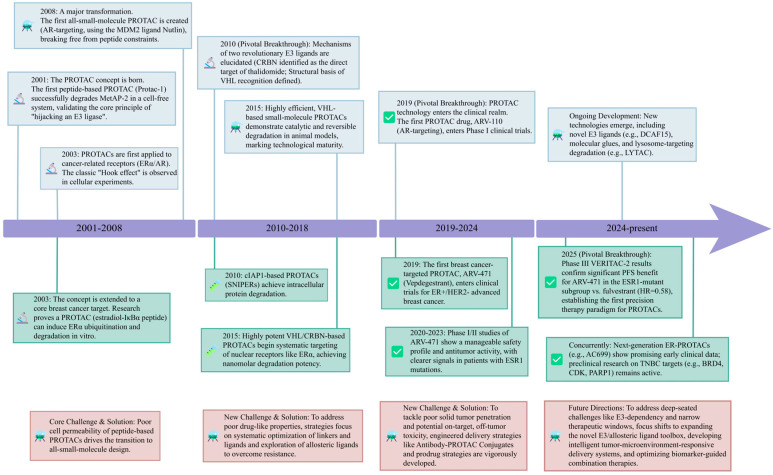
Timeline of the development of PROTAC technology and BC applications: From peptide-based prototypes to small molecule maturation, clinical entry, and the advancement toward patient stratification and delivery engineering.

At the clinical level, the entry of the AR-targeting PROTAC ARV-110 into clinical trials in 2019 marked a pivotal milestone in the translation of PROTACs from a platform technology to therapeutic development [102]. Since then, multiple candidates—including those for indications such as BC—have advanced to clinical-stage evaluation, underscoring the accelerating clinical adoption of TPD strategies (Figure 2).

## 3. Hormone Receptor–Positive/HER2-Negative: Evidence Chain for ER-Axis Targeted Degradation (Mechanism–Medicinal Chemistry–Clinical Stratification)

The ESR1/ERα pathway drives the initiation and progression of ~70% of BC and represents one of the best-validated and most drug-developed receptor axes in BC [3]. Although endocrine therapy has evolved from SERMs and AIs to SERDs, acquired resistance remains common in advanced/metastatic disease [10,11]. Conventional endocrine agents mainly suppress signaling through functional antagonism, whereas PROTACs can reduce ERα protein abundance to achieve a potentially “deeper and more durable” suppression of the ER axis; therefore, they may offer advantages under resistance settings involving receptor conformational changes, compensatory expression, and related adaptive mechanisms [23,24].

Notably, “degradation/downregulation” of ER signaling is not unique to PROTAC-based strategies. Oral SERDs (e.g., elacestrant) have demonstrated clear efficacy in ER-positive/HER2-negative advanced BC previously treated with CDK4/6 inhibitors, with a more pronounced benefit in the ESR1-mutant subgroup. These data support the clinical feasibility and testability of both the “ER degradation/downregulation” mode of action and “ESR1 mutation–based stratification” [103].

Against this backdrop, the potential clinical positioning of ER-targeting PROTACs can be summarized as follows: assuming oral dosing is achievable, PROTACs actively recruit an E3 ligase to form a ternary complex and drive programmable, catalytic ER clearance, thereby providing an effect depth, PK/pharmacodynamic (PD) profile, and potentially distinct resistance trajectories that are partially differentiated from SERDs during resistance evolution [23,24]. Because the ER axis has both well-established standard-of-care comparators (e.g., fulvestrant) and actionable biomarkers (ESR1 mutations) for patient stratification [12,13,103], ER-targeting PROTACs in BC have formed a relatively complete “mechanism–medicinal chemistry–clinical stratification” evidence chain and may serve as a key reference framework for the clinical translation of precision TPD.

### 3.1. Resistance Landscape of the ER Pathway and the Theoretical Advantages of ER Degradation Strategies: From SERDs to PROTACs

ERα is a member of the nuclear receptor superfamily and contains multiple functional domains: the N-terminal A/B region harbors ligand-independent transcriptional activation function (AF-1); the C region is the DNA-binding domain (DBD); the D region is the hinge; and the C-terminal E/F region constitutes the LBD, which contains the AF-2 surface and the key conformational element helix 12 (H12) that regulates receptor activity [104]. Upon binding agonists such as estradiol, H12 undergoes conformational rearrangement and promotes recruitment of coactivators, whereas antagonists such as tamoxifen primarily alter or restrict the H12 conformation and thereby interfere with coactivator binding; however, partial agonism can still occur in a tissue-dependent manner, indicating that pharmacologic output is jointly determined by receptor conformation, coregulator availability, and cellular context [104,105].

SERDs exemplified by fulvestrant induce ERα conformational changes and reduce receptor stability, thereby accelerating receptor turnover. This process involves the ubiquitin-proteasome system, but it is mechanistically distinct from PROTACs, which achieve programmable degradation by actively recruiting an E3 and promoting a productive ternary complex between the target, degrader, and ligase [34,105,106]. Accordingly, although both SERDs and PROTACs can lower ERα protein levels, they are not equivalent in molecular mechanism, PK/PD logic, or potential resistance pathways. SERDs remain largely exposure- and occupancy-dependent: their clinical activity depends on sustained target engagement and sufficiently durable receptor downregulation, and resistance is often linked to mechanisms that reduce effective antagonistic occupancy or limit the depth and duration of ER suppression, including LBD conformational activation, rapid receptor resynthesis, and broader pathway rewiring. By contrast, PROTACs are event-driven degraders: once a productive ternary complex is formed, they can catalytically trigger ubiquitination and proteasomal elimination of ERα, such that pharmacologic output depends not only on binary target binding but also on E3 recruitment, ternary-complex cooperativity, ubiquitin-transfer competence, and adequate intracellular exposure. Therefore, resistance to PROTACs can additionally arise from perturbations of the E3 ligase-ternary complex-UPS axis, including altered E3 ligase availability or function, impaired ternary-complex formation, reduced ubiquitination efficiency, or distribution/efflux constraints that lower effective intracellular degrader concentration [34,106].

In acquired endocrine resistance, gain-of-function ESR1 mutations within the LBD, particularly Y537S and D538G, are especially important because they stabilize H12 in a more agonist-like conformation and confer ligand-independent ERα activity [12,107]. These alterations are uncommon in untreated primary tumors but become enriched in metastatic disease after prolonged endocrine therapy, consistent with clonal selection under treatment pressure [12,107]. This biology helps explain why strategies that rely mainly on LBD occupancy and conformational suppression may encounter a PK/PD ceiling in some endocrine-resistant tumors. By contrast, PROTAC-mediated ER degradation aims to reduce total intracellular ERα abundance and therefore has a stronger theoretical basis for attenuating both ligand-dependent and ligand-independent ER signaling outputs in ESR1-mutant disease [25,105].

This distinction is also important from a biomarker perspective. ESR1 mutations should not be viewed only as resistance biomarkers; in appropriately defined clinical settings, they may also function as predictive biomarkers for strategies aimed at enhanced ER degradation/downregulation. This interpretation is supported by the broader clinical experience with next-generation ER-lowering agents. In the phase III EMERALD trial, the oral SERD elacestrant improved progression-free survival (PFS) over standard endocrine therapy and showed greater benefit in the ESR1-mutant subgroup [103]. More recently, in the phase III VERITAC-2 trial, the ER PROTAC vepdegestrant significantly prolonged PFS versus fulvestrant in patients with ESR1-mutant ER-positive/HER2-negative advanced BC, establishing clinical proof-of-concept that deeper ER degradation can be particularly relevant in this biomarker-defined population [25,108]. Taken together, these data support a more actionable formulation: ESR1 mutation status may help identify endocrine-resistant tumors in which enhanced ER downregulation—not simply renewed receptor occupancy—is more likely to provide net benefit. At the same time, this predictive interpretation should not be overgeneralized, because clinical benefit remains dependent on treatment line, co-occurring bypass alterations, and residual ER dependence of the tumor [25,103,105].

**Figure 3:**
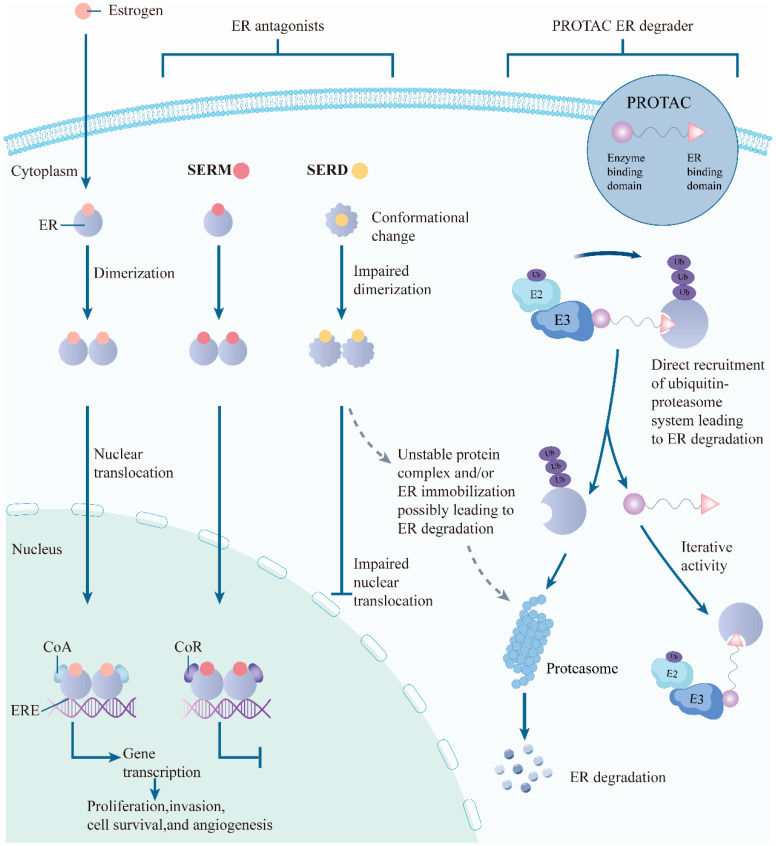
Schematic illustration of ER signal transduction and the mechanisms of different endocrine intervention strategies: Traditional antagonism/modulation vs. PROTAC-mediated targeted ER degradation.

ESR1 mutations are thus increasingly shifting from a mechanistic explanatory variable to a clinical decision variable. The prospective randomized PADA-1 study showed that, during palbociclib plus AI therapy, early treatment adaptation triggered by rising ESR1 mutation levels in circulating tumor DNA (ctDNA) yielded clinically meaningful benefit [30]. The translational implication is that ER-targeting degraders, including oral SERDs and ER PROTACs, are most likely to maximize net benefit when integrated into a dynamic strategy of molecular monitoring, resistance stratification, and timely therapeutic switching rather than simply substituted into an unselected population. Guided by this clinical problem orientation, the next section will further outline how ER PROTACs increase degradation depth while attempting to preserve druggability, and will provide a mechanistic bridge to the later discussion of PK/PD, resistance, and therapeutic window. These intervention modes are schematically compared in Figure 3.

### 3.2. Medicinal-Chemistry Evolution of ER PROTACs: Linker Attachment/Linker Optimization and Expansion of E3-Recruitment Strategies

ERα was among the earliest canonical model targets for PROTAC development, and its trajectory clearly illustrates the key steps by which the technology progressed from proof-of-concept to drug-like optimization. Early efforts largely relied on peptide-based or peptide–small-molecule hybrid PROTACs. In 2003, a molecule created by conjugating an IκBα phosphopeptide (recruiting SCF^β-TRCP) to estradiol provided the first cell-free demonstration that ER ubiquitination and proteasome-dependent degradation could be induced; a typical hook effect was also observed, thereby establishing—at the mechanistic level—the framework that “ternary-complex formation drives degradation” [91]. Subsequently, a VHL-recognized peptide–estradiol PROTAC achieved intracellular ER degradation, extending the evidence to cellular systems [109]. Although poor membrane permeability and limited in vivo stability hindered translation of peptide-based designs, this phase made a central point explicit: the ternary-complex geometry shaped by the linker attachment site and linker architecture is a core determinant of degradation output.

With ternary-complex cooperativity and drug-likeness as guiding principles, engineering of attachment sites and linkers gradually converged on reusable design rules. The linker exit vector can be decisive for activity; for example, conjugation at estradiol C-7α often outperforms that at the O-17 position [89]; linker length also exhibits an “optimal window”, as excessively short or long linkers may markedly reduce degradation due to geometric mismatch [80]. In addition, multivalency strategies (e.g., “bivalent/two-headed PROTACs”) can enhance degradation by increasing local effective concentration and promoting ternary-complex formation [110]. Accordingly, optimization of ER PROTACs should not focus solely on binary affinity at each end, but rather prioritize ternary-complex cooperativity, the intracellular effective concentration window, and the coupling between Dmax/DC_50_ (degradation depth/duration) and phenotypic outputs [66,76,77,78,79].

At the molecular modality level, ER degraders underwent a key transition from peptide-based constructs to fully small-molecule designs. On the one hand, approaches such as stabilized cell-penetrating peptides improved membrane permeability and in vivo performance of peptide PROTACs, providing methodological groundwork for later expansion to non-canonical binding interfaces [111,112]. On the other hand, the move to fully small-molecule degraders substantially increased druggability. In 2011, the first SNIPER(ER), built from the cIAP1 ligand bestatin and estrone, achieved intracellular ERα degradation [97]; its derivative SNIPER(ER)-3 triggered, in addition to degradation, reactive oxygen species (ROS)-dependent cell death—suggesting that degraders may drive cell-fate outputs distinct from simple antagonism, while also implying a more complex effect spectrum that requires rigorous separation of “target-dependent degradation effects” from “scaffold-associated non-specific stress effects” in follow-up studies [98,99].

As high-affinity small-molecule E3 ligand systems such as VHL and CRBN matured, ER-targeting degraders achieved a marked leap in potency. The VHL-recruiting ERD-308 reached sub-nanomolar potency against ERα (DC_50_ ≈ 0.17 nM), with degradation kinetics and anti-proliferative activity superior to those of Fulvestrant [94]; ERD-148 efficiently cleared phosphorylated ERα species that are relatively insensitive to Fulvestrant and retained clearance capability in ESR1-mutant contexts, thereby mechanistically supporting the notion that “deeper receptor removal can cover scenarios in which SERDs insufficiently address specific receptor states” [93].

Meanwhile, under endocrine-resistance pressure, design concepts for ER degraders have begun to expand from “continued reliance on LBD ligands” toward “diversified binding interfaces and pathway nodes”. On the one hand, most oral ER PROTACs still use LBD ligands as the targeting warhead, but their advantage lies in maintaining binding while enabling protein-level elimination of mutant receptors, thereby attenuating the impact of “mutation-driven loss of antagonist potency” [113]. On the other hand, given the risk that LBD mutations may further evolve, alternative recognition interfaces are being explored. For example, nucleic-acid–conjugated ERE PROTACs use an estrogen response element (ERE) sequence as the recognition unit to instead engage the ERα DBD and induce proteasome-dependent ERα degradation in cells—providing a proof-of-concept route to bypass LBD mutations [27].

Beyond the receptor itself, key coactivators within the ER pathway act as network hubs in endocrine resistance. SRC3 plays a pivotal role in ER signaling output and endocrine resistance; a recently reported SRC3 PROTAC (using SI-2 as the targeting ligand and recruiting CRBN) efficiently degraded SRC3 and produced pronounced anti-proliferative effects in ER-positive and ESR1-mutant resistant models, indicating that “degradation of coactivators” can serve as a complementary strategy beyond direct ER degradation [28]. Similarly, a PROTAC that recruits a UBR-box-domain E3 ligase achieved selective degradation of SRC1 and suppressed invasion and migration, offering another feasible route for “E3 diversification + pathway-node migration” [101].

“Dual-targeting/multivalent” designs also represent a direction to deepen pathway suppression and reduce bypass escape. For instance, a PROTAC that simultaneously targets ERα and aromatase (ARO) achieved concurrent ERα/ARO degradation in vitro and in vivo and inhibited tumor growth; it also retained degradation and anti-proliferative activity in ER-mutant cells, suggesting potential resistance-suppressing value for a combined clearance strategy integrating “receptor signaling plus ligand supply” [29]. The design evolution of ER-targeting PROTACs is summarized in Table 3.

### 3.3. Vepdegestrant (ARV-471): From Preclinical Mechanistic Validation to Biomarker-Guided Translation

Vepdegestrant is among the most clinically advanced ER-targeting PROTACs to date. It is a CRBN-recruiting degrader assembled by tethering an optimized ER ligand to a pomalidomide-derived CRBN binder through a linker, and it has oral dosing potential [31]. Its preclinical package is highly translational, as it systematically addresses three key clinical questions: (i) whether degradation is the dominant pharmacological mechanism, (ii) whether activity is retained in ESR1 mutation–driven endocrine resistance, and (iii) whether the agent provides a scalable foundation for combination therapy.

At the mechanistic and PD level, vepdegestrant induces robust degradation of both wild-type ERα and multiple ESR1-mutant ERα variants in cellular models, with maximal degradation exceeding 95%. By benchmarking against a control compound that binds ER but fails to productively recruit CRBN, the antiproliferative activity of vepdegestrant was shown to be explicitly degradation-dependent, thereby causally separating “binding/antagonism” from “degradation” and reinforcing its mechanistic identity as a PROTAC rather than a modified ER antagonist. In patient-derived xenograft (PDX) models, orally administered vepdegestrant achieved deep and relatively durable ER depletion and produced tumor growth inhibition; these effects were superior to those of fulvestrant across multiple models, with a more pronounced advantage in ESR1 Y537S–mutant tumors [31]. Collectively, these findings support the concept that, in resistance settings where ER remains a key driver but conventional endocrine therapies have diminished efficacy, deeper ER removal may translate into a higher maximal pharmacological effect.

With respect to combination strategies, vepdegestrant showed synergistic activity with cyclin-dependent kinase 4/6 (CDK4/6) inhibitors in preclinical models, enabling concurrent suppression of upstream receptor signaling and downstream cell-cycle machinery [31]; synergy was also observed with phosphoinositide 3-kinase alpha (PI3Kα) or mTOR inhibitors, providing a mechanism-consistent rationale to counteract pathway crosstalk and compensatory activation between ER signaling and PI3K/AKT/mTOR bypass circuits [31]. Notably, clinical feasibility of such combinations depends not only on mechanistic synergy, but also on achievable exposure, overlapping toxicities, and optimization of dosing schedule; these considerations are summarized in Section 6.3.3.

From a translational perspective, the key implication of vepdegestrant is that the net clinical benefit of ER PROTACs is more likely to be realized through biomarker-driven stratification rather than as a uniform advantage in unselected populations. The pivotal findings, statistical framework, safety profile, and population heterogeneity of the phase III VERITAC-2 trial are discussed in detail in Section 6.2.1 [25]. Mechanistically, tumors harboring ESR1 mutations more closely align with the biological premise of sustained ER-dependent output driven by an activated receptor conformation; therefore, deeper ER depletion may be more likely to yield an observable efficacy differential in this subgroup. In contrast, among heavily pretreated ESR1 wild-type patients, progression may be more frequently shaped by bypass signaling and tumor heterogeneity (e.g., PI3K/MAPK pathway alterations) [14], in which case intensifying ER depletion does not necessarily translate into a PFS advantage.

Importantly, oral SERDs have also demonstrated clinical benefit in patients previously treated with CDK4/6 inhibitors, particularly in those with ESR1 mutations [103]. In this context, an expert commentary highlighted that, based on VERITAC-2, vepdegestrant demonstrated improved PFS versus fulvestrant in the prespecified ESR1-mutant subgroup, raising a practical question that is increasingly shifting from ‘whether ER lowering works’ to ‘which ER-lowering strategy (oral SERD versus ER-targeting PROTAC) should be selected and optimally sequenced in endocrine-resistant ER-positive/HER2-negative disease’ [108]. The same commentary also highlighted a real-world positioning limitation of VERITAC-2: patients previously treated with fulvestrant were excluded, leaving the activity of vepdegestrant after prior fulvestrant (or after other SERDs) as an unresolved sequencing issue with direct implications for treatment algorithms [108]. This perspective complements and extends the PADA-1 paradigm of dynamic ctDNA monitoring of ESR1 mutations to guide treatment adaptation [30], supporting the positioning of ER PROTACs within a framework of dynamic stratification, mechanism matching, and combination/sequencing optimization.

Finally, as a CRBN-recruiting degrader, vepdegestrant warrants continued attention to potential acquired resistance driven by alterations in the CRBN axis, as well as the risk of unintended neosubstrate degradation associated with immunomodulatory imide (IMiD)-based recruiters. These issues provide important context for the discussion of resistance mechanisms (Section 6.2.1) and the “exposure–selectivity–therapeutic window” constraint in solid tumors (Section 6.3) [81,82,83]. Taken together, vepdegestrant exemplifies a translational logic in which deep ER depletion serves as the mechanistic foundation, ESR1 mutations and other dynamically monitorable biomarkers serve as stratification entry points, and optimization of combination/sequencing strategies becomes a key lever to expand the net-benefit window.

## 4. HER2-Positive BC: Antigen-Mediated Delivery Strategies to Improve the Therapeutic Window of PROTACs

HER2-positive BC accounts for approximately 15–20% of all BC cases and is characterized by HER2 gene amplification and/or protein overexpression, which drives sustained activation of downstream pro-survival and proliferative signaling and is often associated with a more aggressive clinical phenotype [39,40]. Anti-HER2 therapies, including trastuzumab, pertuzumab, and ADCs, have markedly improved patient outcomes; nevertheless, acquired resistance remains common. Reported mechanisms include alterations of the HER2 receptor itself, activation of bypass pathways, and compensatory upregulation of downstream signaling such as the PI3K/AKT/mTOR axis [16]. Translational experience from HER2-targeted ADCs has underscored that resistance and variable benefit are jointly shaped by antigen heterogeneity/downregulation, internalization/trafficking efficiency, intracellular processing, and payload-related adaptations. These insights have motivated rational combination strategies while emphasizing tolerability constraints, and they provide a valuable conceptual framework for the development of HER2-guided Ab–PROTACs, nanocarriers, and combination regimens [5]. In this context, the potential value of TPD in HER2-positive BC may be viewed from two complementary angles: (1) by reducing the abundance of the receptor or key signaling nodes, degraders may achieve deeper and potentially more durable pathway suppression than inhibitors, thereby attenuating adaptive rewiring following receptor tyrosine kinase (RTK) blockade; and (2) by leveraging HER2 as a cell-surface internalization gateway, antibodies, nanocarriers, or aptamers can be used to enable selective delivery of PROTAC payloads, increasing intratumoral effective exposure while reducing toxicity associated with systemic exposure, ultimately expanding the therapeutic window.

Compared with the ER-positive setting, translational progress for PROTACs that directly target HER2 has been slower, largely due to target biology and solid-tumor pharmacology constraints. First, HER2 is also expressed in normal tissues, and existing anti-HER2 therapies have already highlighted the need for cardiac safety vigilance; deeper and sustained HER2 protein removal could amplify “on-target, off-tumor” risk and narrow the therapeutic window. Second, because HER2 is a transmembrane receptor, its membrane localization, endocytic trafficking, and dimerization dynamics with partners such as HER3 may influence whether and to what extent it can be efficiently accessed and degraded via the UPS. Together with the inherently high molecular weight of PROTACs and their limitations in permeability and tumor distribution, these factors make HER2-positive solid tumors particularly dependent on delivery-driven, cell-selective degradation, rather than simply maximizing in vitro degradation potency. Accordingly, this section proceeds from proof-of-concept studies for direct HER2 degradation to HER2-guided delivery platforms, and then to the degradation of downstream resistance nodes, highlighting the pivotal role of delivery strategies in HER2-positive disease.

### 4.1. Direct HER2 Degradation: Proof-of-Concept Evidence, the Boundaries of Membrane-Protein Degradation, and Therapeutic-Window Constraints

The rationale for directly degrading HER2 is to remove the receptor itself, thereby simultaneously abrogating its kinase activity and potential scaffold functions, achieving more complete—and potentially more durable—signal suppression than inhibition alone, and reducing compensatory pathway rebound. Burslem et al. constructed a VHL tumor suppressor–recruiting PROTAC using lapatinib as the targeting “warhead” (Compound 1) and compared it with a stereoisomeric control (Compound 2) in which the VHL ligand stereochemistry was altered to abolish productive VHL recruitment [35]. In SKBr3 cells, Compound 1 reduced EGFR and HER2 protein levels and showed stronger antiproliferative activity (72 h IC_50_: 102 nM; Compound 2: 171 nM). At the same saturating concentration (500 nM), degradation (vs. inhibition) produced more sustained downstream signaling suppression and attenuated kinase-network reprogramming/bypass rebound within 24–48 h, including extracellular signal-regulated kinase (ERK), AKT, and HER3/c-Met–associated signaling. This work provides a key proof-of-concept that RTK membrane proteins can, at least in certain contexts, be degraded via a canonical PROTAC mechanism [35].

However, canonical PROTACs primarily rely on the UPS, which is most efficient at eliminating cytosolic and nuclear proteins, whereas the turnover of many transmembrane receptors is dominated by the endocytosis–lysosome route. Consequently, whether UPS-based strategies can deliver stable, reproducible, and generalizable degradation of membrane proteins remains context-dependent and uncertain across targets and cellular backgrounds [41,42,43].

Moreover, the evidence above is largely cell-based and is not yet supported by systematic in vivo exposure–distribution–toxicity datasets [35]. Importantly, current HER2-degrader evidence is still largely cell-line–based and lacks matched in vivo tumor PK/intratumoral PD and systematic safety characterization, limiting confidence in therapeutic-window feasibility at this stage. From a translational standpoint, the central limitation of directly degrading HER2 is less “whether HER2 can be degraded” and more “whether HER2 can be degraded selectively and controllably within tumors”. On the one hand, HER2 expression in normal tissues creates systemic toxicity pressure; on the other hand, membrane localization and endocytic trafficking dynamics add additional barriers to achieving robust degradation in solid tumors. Accordingly, the research focus on HER2-positive disease has gradually shifted from “HER2 as a direct PROTAC target” toward “HER2 as a receptor-mediated endocytosis anchor” for targeted delivery, using delivery engineering to expand the therapeutic window.

### 4.2. HER2-Mediated, Cell-Selective Delivery: Ab–PROTACs, Nanocarriers, and Aptamer–PROTAC Platforms

In HER2-positive BC, a more translationally actionable “delivery-driven” paradigm is to use HER2 as a tumor-cell surface antigen to enable selective binding and receptor-mediated endocytosis via antibodies or other ligands, followed by intracellular release of a PROTAC to degrade key oncogenic or maintenance nodes (e.g., BRD4, RIPK2). Conceptually, this strategy shifts selectivity upstream from “molecular binding to the POI” to “cellular recognition and uptake of a tumor-associated antigen”, which may reduce the impact of systemic exposure on normal tissues and provide a more practical therapeutic window for intracellular targets whose tumor/normal expression differences are insufficient.

A representative modality is the antibody–PROTAC conjugate (Ab–PROTAC). Investigators conjugated a PROTAC targeting bromodomain-containing protein 4 (BRD4) to trastuzumab via an intracellular protease-cleavable linker to generate Ab–PROTAC3 [36]. This construct operates via a stepwise control mechanism: trastuzumab mediates HER2-selective binding and internalization; upon entry, the linker is cleaved in the lysosomal/intracellular milieu to release the active PROTAC; the liberated PROTAC then induces BRD4 degradation in the cytosol/nucleus. Experimentally, this conjugate triggered BRD4 degradation only in HER2-positive cells, with no such effect in HER2-negative cells, and imaging/trafficking data supported the sequence of “HER2-mediated endocytosis → lysosomal trafficking → payload release → target degradation” [36]. Notably, the lysosome primarily serves as a delivery and controlled-release compartment, whereas the released PROTAC still degrades BRD4 mainly through the UPS in the cytosol/nucleus; therefore, cytosolic accessibility/escape efficiency of the payload after endocytosis is often a key engineering determinant of degradation performance [41,42,43].

This platform is extendable to additional targets. When an Ab–PROTAC was used to deliver a PROTAC against receptor-interacting serine/threonine-protein kinase 2 (RIPK2), substantial RIPK2 degradation was observed in HER2-positive cell lines; replacing trastuzumab with a non-HER2-binding control antibody abolished the degradation effect. In HER2-negative cells, neither construct produced effective degradation, further supporting that cell selectivity is primarily dictated by surface-antigen recognition [95]. Translationally, these studies highlight that HER2 is not only a therapeutic target to inhibit but also a delivery anchor that can enable more controlled tumor-cell entry of PROTAC payloads, thereby expanding TPD to a broader range of intracellular oncoproteins.

Nanocarriers can likewise improve effective delivery and intracellular exposure in solid tumors. For example, the BRD4 degrader MZ1 was encapsulated into nanoparticles and actively targeted using trastuzumab to generate MZ1–ACNPs; this system increased drug accumulation and cytotoxicity in HER2-positive cells and, in some models, overcame intrinsic resistance to free MZ1 [37]. These results suggest that, in HER2-positive solid tumors, resolving distribution, cellular uptake, and intratumoral effective exposure may be more decisive for in vivo efficacy than further maximizing in vitro degradation potency.

“Functionally relevant” surface proteins may also serve as delivery/degradation entry points. Nucleolin (NCL) is aberrantly expressed on the surface of multiple cancer cell types and has been reported to functionally interact with ErbB2. Clinically, NCL overexpression is associated with poorer survival in ErbB2-positive BC, and NCL inhibition reduces the viability of ErbB2-positive cancer cells and attenuates ErbB2 activation [114]. On this basis, an aptamer-based PROTAC was developed by using AS1411 as an NCL-targeting aptamer and conjugating it to a VHL ligand to generate ZL216, thereby exploiting differential NCL expression for selective binding and internalization and inducing NCL degradation in vitro and in vivo, ultimately suppressing BC cell proliferation and migration [38]. This “aptamer–PROTAC” strategy further reinforces the central theme of this section: in HER2-positive solid tumors, delivery/uptake and cell selectivity often determine translational feasibility before the question of “whether degradation is possible”.

In addition, for certain BC subgroups that are HER2-positive or exhibit signaling crosstalk with HER2, degrading alternative pathway-dependent nodes may be a complementary approach. For instance, the AR degrader ARD-61 showed stronger antiproliferative activity than antagonists and achieved in vivo degradation in AR-positive BC models [115]. Whether such an approach applies to HER2-positive populations will depend on co-expression patterns and clinically verifiable biomarker-based stratification.

Overall, the goal of these strategies is to use the HER2 antigen to confine payload exposure as much as possible to tumor cells, followed by catalytic clearance of intracellular POI by PROTACs. Key next-step questions include linker stability and controllable release, cytosolic escape efficiency after endocytosis, and the impact of HER2 expression heterogeneity on delivery uniformity [41,42,43].

### 4.3. PROTAC-Mediated Degradation of Downstream Resistance Nodes in HER2 Signaling: Resensitization via the PI3K–AKT–mTOR and Transcriptional CDK Axes

When resistance to anti-HER2 therapy arises from compensatory activation of downstream pathways, an alternative and potentially tractable approach is to use PROTACs to directly degrade key downstream signaling hubs, thereby functionally “bypassing” receptor-level resistance. The PI3K–AKT–mTOR pathway is a core pro-survival axis downstream of HER2, and its aberrant activation—often associated with PIK3CA and/or phosphatase and tensin homolog (PTEN) alterations—is a major mechanism of resistance to anti-HER2 therapy. Although PIK3CA mutations are more frequent in hormone receptor-positive/HER2-negative BC, their biological and clinical relevance in resistance within HER2-positive BC is also well established [44].

In this context, a PI3K p110α isoform-selective PROTAC was developed. In lapatinib-resistant cell models and patient-derived organoids, this degrader efficiently eliminated p110α, induced G1-phase arrest, and, at lower concentrations, resensitized resistant cells and restored responsiveness to lapatinib, with overall effects superior to those of the p110α inhibitor alpelisib [16]. These findings suggest that, in resistance states driven by sustained pathway hyperactivity and/or persistently elevated protein abundance, physical removal of the protein may deliver more complete pathway suppression than enzymatic inhibition, thereby increasing the likelihood of reversing resistance.

Beyond the PI3K axis, CDK9, a transcriptional regulatory kinase, has been proposed as a vulnerability in subsets of HER2-positive BC (particularly ER-positive/HER2-positive disease) and in models of resistance to HER2-targeted therapy. The PROTAC THAL-SNS-032 induces CDK9 degradation and demonstrates potent antiproliferative activity in ER-positive/HER2-positive cells and in HER2-targeted therapy–resistant models, with higher overall efficacy than its parent kinase inhibitor [15]. However, the same study also indicated that systemic, non-selective, and highly potent degradation may cause substantial toxicity (e.g., gastrointestinal toxicity), resulting in a narrow therapeutic window. Thus, for broadly expressed targets with essential physiological functions, “strong degradation” does not necessarily translate into “clinical tractability”, and delivery selectivity and exposure control remain decisive determinants [15].

Overall, PROTACs’ applications in HER2-positive BC place particular emphasis on delivery and selectivity control. Moreover, as HER2 is a transmembrane receptor, achieving efficient, selective, and controllable UPS-mediated degradation within solid tumors is often more challenging. In this regard, complementary membrane-protein degradation technologies that preferentially engage lysosomal pathways—such as lysosome-targeting chimeras (LYTACs) and antibody-based targeted chimeras (AbTACs)—represent promising directions to broaden HER2-relevant targeted degradation strategies [116,117]. Representative HER2-positive BC PROTAC strategies are summarized in Table 4.

## 5. TNBC: PROTAC Target Landscape and Evidence Types in the Context of Heterogeneity

TNBC lacks expression of ER, progesterone receptor (PR), and HER2, and therefore shows limited response to endocrine therapy or anti-HER2 regimens. It is a BC subtype associated with poor prognosis and limited therapeutic options [4,48]. TNBC is characterized by marked genomic and phenotypic heterogeneity and by the absence of stable, pathognomonic driver targets; consequently, chemotherapy remains the cornerstone of systemic treatment, yet resistance and treatment-related toxicities substantially constrain survival benefit [17,46,118]. TPD technologies such as PROTACs do not rely on the catalytic activity of the POI and can eliminate key components that are traditionally difficult to inhibit, including transcription factors and scaffolding proteins, thereby offering new intervention entry points for TNBC. Given that TNBC is not a single disease entity but comprises multiple molecular subgroups [47], its vulnerabilities may arise from distinct dependencies, such as homologous recombination repair deficiency, MYC overexpression, or dysregulated cell-cycle control [119,120]. Below, we summarize evidence for TNBC-relevant PROTACs by functional target class, covering transcriptional/epigenetic regulation, DNA damage repair, the cell cycle and transcriptional kinases, invasion–metastasis and scaffolding proteins, as well as tumor-suppressive and apoptosis-regulatory pathways.

### 5.1. Transcriptional/Epigenetic Dependencies: BET/BRD4, MYC, AR, and PRMT5 as Actionable Nodes in TNBC

Transcriptional and epigenetic dysregulation represent a recurrent vulnerability layer in TNBC, but the specific dependency varies by molecular context and subtype. Accordingly, this section summarizes TNBC-relevant TPD evidence across four parallel modules: (i) BET/BRD4-centered chromatin-reader dependence (including delivery-enabled amplification and E3-ligase–dependent combination behavior), (ii) MYC-oriented PROTAC-like nucleic-acid strategies, (iii) AR-positive/luminal androgen receptor (LAR) TNBC as a nuclear receptor–driven transcriptional dependency state, and (iv) additional epigenetic regulators exemplified by PRMT5.

#### 5.1.1. BET/BRD4 Degradation and Delivery-Enabled Amplification

Bromodomain and extra-terminal (BET) family proteins (exemplified by BRD4) sustain oncogenic transcriptional programs by recognizing acetylated histones and organizing transcriptional complexes, thereby supporting a “transcriptionally addicted” phenotype. In BC samples and cohorts, including TNBC, evidence supports BET-associated gene expression signatures and upregulation of BRD2/BRD3/BRD4 proteins [121].

Unlike BET inhibitors, which primarily block bromodomain–acetyl-lysine recognition, BRD4 degradation removes the protein itself and concomitantly abrogates its scaffold functions, often yielding more coherent suppression at both the transcriptional and phenotypic levels. The BET degrader BETd-246 rapidly eliminated BRD4 at nanomolar concentrations and outperformed its parent inhibitor in suppressing TNBC cell proliferation and inducing apoptosis, while downregulating gene networks implicated in proliferation and apoptosis evasion. Transcriptomic comparisons further indicated that BET inhibition frequently produced mixed up- and down-regulated responses, whereas degradation more consistently drove dominant downregulation of proliferation/survival networks [50]. Notably, “degradation-over-inhibition” conclusions can be context-dependent because exposure, intracellular free concentration, and off-target liabilities are rarely matched across comparator molecules, and thus require careful control-based interpretation.

ARV-825 also showed activity in TNBC and resistant settings; notably, MZ1 and ARV-825 continued to reduce BRD4 abundance and induced cell-cycle arrest and caspase-dependent apoptosis in TNBC models, including those with resistance to BET inhibitors, supporting the feasibility of shifting from “inhibition” to “degradation” to overcome resistance [49].

Because pharmacokinetics (PK) and tissue distribution can limit in vivo efficacy, delivery systems may be leveraged to amplify antitumor activity and improve the therapeutic window. Liposomal co-delivery of ARV-825 and docetaxel improved solubility and tumor accumulation and enhanced tumor growth inhibition in the 4T1 model, accompanied by immunogenic cell death and increased CD8+ T-cell infiltration [65].

In addition, distinct E3 ligase recruitment modes can generate different transcriptional perturbations and combination-therapy compatibility profiles. A VHL-recruiting BRD4 PROTAC induced more pronounced mRNA expression changes in TNBC and showed synergy with cisplatin but antagonism with paclitaxel, suggesting that rational combinations should be guided by empirical evidence rather than directly extrapolated from inhibitor-based experience [96]. Recent work further suggested that increasing BRD4 selectivity over BRD2/BRD3 while concomitantly suppressing super-enhancer–driven core transcription factors (e.g., Krüppel-like factor 5 (KLF5)) may further strengthen inhibitory effects in basal-like BC and a subset of TNBC models [122].

BET/BRD4 degradation serves as a prototypical chromatin-reader TPD strategy in TNBC, whereas formulation/delivery and E3-ligase choice materially shape both pharmacology and combination behavior.

#### 5.1.2. MYC-Oriented PROTAC-like Strategies Enabled by Oligonucleotides/Aptamers

For the transcription factor c-Myc, which remains challenging for conventional small-molecule drug discovery, oligonucleotide-/aptamer-enabled PROTAC-like strategies have been explored to achieve targeted degradation of c-Myc–related complexes and to enhance TNBC sensitivity to CDK4/6 inhibitors. In these designs, nucleic-acid ligands (e.g., threose nucleic acid (TNA) aptamers or E-box DNA binding sequences) replace the small-molecule targeting “warhead.”

Prior studies demonstrated selective intracellular degradation of the endogenous c-Myc/Max complex, and combination with palbociclib produced stronger tumor growth suppression in murine TNBC models [52]. However, such systems are more dependent on delivery, stability, and cellular uptake; therefore, translation typically requires integration with nanodelivery platforms, local administration, or TME–responsive designs.

This module targets a core oncogenic transcription factor complex, complementing BET/BRD4 degradation mechanistically, but its translational bottlenecks shift more toward delivery and in vivo stability.

#### 5.1.3. AR-Positive TNBC (LAR Subtype): A Nuclear Receptor–Driven Transcriptional Dependency and AR Degradation

In addition to chromatin- and MYC-linked transcriptional addiction, a clinically relevant subset of TNBC exhibits AR expression and an AR-associated transcriptional program, commonly referred to as the luminal androgen receptor (LAR) subtype [123,124]. Rather than being subsumed within unselected TNBC, AR-positive/LAR disease merits separate consideration because it represents a biologically distinct, nuclear receptor-driven subgroup with potentially different therapeutic vulnerabilities and, in several series, lower chemosensitivity than other TNBC states [124]. AR-positive/LAR TNBC thus represents a nuclear receptor–driven transcriptional dependency state that is intrinsically biomarker-enriched.

Clinical evidence supports AR as an actionable target in this subset: a phase II study of the AR antagonist bicalutamide reported clinical benefit in ER-negative/AR-positive BC [125]. Subsequent anti-androgen studies broadened this signal: abiraterone plus prednisone produced a 6-month clinical benefit rate of 20.0% with a median PFS of 2.8 months in AR-positive TNBC [126], and enzalutamide demonstrated a 16-week clinical benefit rate of 25% in the intention-to-treat population and 33% in the evaluable subgroup, indicating that AR blockade has modest but reproducible activity in biomarker-selected advanced disease [127]. However, heterogeneous responses and incomplete pathway suppression with occupancy-driven antagonism motivate evaluation of deeper pathway shutdown strategies. These results also suggest that AR-directed therapy is more likely to succeed when applied to biologically enriched disease rather than to unselected TNBC populations.

More recent studies further emphasize that patient selection is critical. In TBCRC 032, enzalutamide combined with taselisib yielded a higher clinical benefit rate than historical single-agent AR blockade [128], and correlative analyses suggested that AR immunohistochemistry alone may be insufficient to define true AR dependency; instead, luminal androgen receptor biology and related molecular features may better identify responsive tumors [128]. In the neoadjuvant setting, enzalutamide plus paclitaxel in LAR-enriched TNBC achieved the prespecified efficacy signal, with 42% of patients reaching pathologic complete response or residual cancer burden class I [129]. Most recently, the UCBG 3-06 START study with darolutamide further underscored that transcriptomic or functional stratification may outperform AR staining alone for identifying androgen-driven disease [130].

TPD offers such an approach by eliminating AR protein itself. In preclinical BC models, the AR PROTAC degrader ARD-61 induced robust AR depletion and demonstrated stronger antiproliferative and antitumor activity than conventional AR antagonism [115]. Taken together, these data position AR-positive/LAR TNBC as a non-negligible subtype within TNBC in which anti-androgen therapy, rational combination strategies—particularly with PI3K/AKT-pathway inhibition—and next-generation AR degraders may all have therapeutic relevance.

Collectively, AR degradation represents a subtype-informed TPD opportunity within TNBC, complementing BET- and MYC-oriented strategies in a heterogeneity-aware framework.

#### 5.1.4. Additional Epigenetic Regulators: PRMT5 Degradation as an Actionable TNBC Vulnerability

Beyond the BET/MYC/AR axes, epigenetic and transcriptional regulators such as protein arginine methyltransferase 5 (PRMT5) also represent actionable vulnerabilities in TNBC. A CRBN-dependent PRMT5 degrader reduced PRMT5 abundance and exhibited antitumor activity in TNBC cell lines, patient-derived organoids, and xenograft models [51].

Collectively, these findings suggest that degradation of epigenetic targets may be amplified by concomitant suppression of key transcriptional programs (including KLF5-associated networks), and they provide a rationale for subsequent stratification based on transcriptional dependency states [51].

Such “epigenetic enzyme” degraders expand the target landscape beyond chromatin readers, transcription factors, and nuclear receptors, aligning well with biomarker-driven stratification concepts in heterogeneous TNBC.

### 5.2. The DDR Axis: PARP1 Degradation and the CDK12/13–Cyclin K Transcriptional DDR Node

The DNA damage response (DDR) axis comprises an integrated network that enables cells to sense DNA lesions, transmit damage signals, activate repair programs, and ultimately determine cell fate. This network includes both protein nodes that directly execute repair (e.g., poly(ADP-ribose) polymerase 1 (PARP1)) [131] and transcriptional regulatory nodes that indirectly sustain repair capacity by maintaining DDR gene expression (e.g., CDK12/13 in complex with Cyclin K) [132,133]. Accordingly, this section discusses TPD strategies from two complementary perspectives: the “repair execution layer” (PARP1) and the “DDR transcriptional supply layer” (CDK12/13–Cyclin K).

PARP1 is a key factor in DNA single-strand break repair, and PARP inhibitors can elicit synthetic lethality in tumors with homologous recombination deficiency (HRD) [131]. In TNBC, PARP1 is frequently overexpressed [53]. Clinical specimens also indicate that PARP1 expression is higher in invasive ductal carcinoma than in normal breast tissue and is associated with receptor-negative status [53], supporting further development of PARP-axis TPD strategies in TNBC, particularly in HRD-enriched subsets. Within the DDR framework, PARP1 can be viewed as a critical initiator/executor node; thus, changes in its protein abundance may directly modulate the intensity of DNA damage processing and downstream death outcomes.

Converting PARP inhibitors into PROTACs can advance the modality from “inhibition” to “elimination”, thereby enhancing efficacy and potentially extending coverage to resistance contexts. A PARP1 PROTAC derived from niraparib induced stronger apoptosis and growth inhibition in TNBC cell lines, while exhibiting lower cytotoxicity toward non-malignant cells [134]. Evidence for the PARP1 PROTAC NN3 further highlights mechanistic gains conferred by degradation: in p53-positive cancer cells, NN3 triggered ferroptosis by downregulating the solute carrier family 7 member 11 (SLC7A11) pathway, and it retained PARP1-degrading activity against PARP1 variants harboring resistance-associated mutations, offering a potential strategy to address loss of inhibitor potency caused by binding-site mutations [135]. Collectively, the value of PARP1 degradation extends beyond “stronger inhibition”; by removing the protein, it may rewire DDR signaling and cell-death modalities, creating additional entry points for second-line strategies and rational combinations after resistance [131].

Beyond PARP-centered interventions, transcriptional maintenance of DDR genes itself can constitute a therapeutic vulnerability. The transcription-associated kinases CDK12/13 form complexes with Cyclin K, contribute to phosphorylation of Ser2 on the C-terminal domain (CTD) of RNA polymerase II (RNA Pol II), and support transcription and co-transcriptional processing of a subset of DDR genes [132,133]. Here, this module is better conceptualized as a “transcription-coupled DDR node”. The CDK12-selective degrader BSJ-4-116 selectively eliminated CDK12 and induced premature cleavage and polyadenylation of DDR transcripts, showing marked antiproliferative activity as monotherapy and in combination with olaparib [55]. In TNBC-focused studies, the selective CDK12 degrader PP C8 suppressed DDR gene transcription and enhanced the antitumor effects of PARP inhibitors, supporting a combination strategy of “transcriptional DDR-node degradation plus synthetic lethality” [54]. Moreover, dual CDK12/13 degraders achieved low-nanomolar degradation in TNBC cells and reduced expression of core DDR genes; compared with their corresponding inhibitors, they exhibited stronger antiproliferative activity, suggesting that the “degradation-over-inhibition” advantage along this axis warrants further validation [136].

Overall, PARP1 degradation targets the DDR “repair execution layer”, whereas CDK12/13–Cyclin K degradation primarily impairs repair capacity by weakening DDR gene expression. Together, these approaches constitute TPD strategies that intervene in the DDR axis at distinct hierarchical levels.

### 5.3. CDKs: Efficacy–Toxicity Trade-Offs of CDK4/6 and CDK9 Degradation and the Need for Prodrug Strategies

CDKs are central regulators of cell-cycle progression and transcriptional homeostasis. Clinically, CDK4/6 inhibitors (e.g., palbociclib) are primarily used in ER-positive BC, where efficacy depends on an intact retinoblastoma (Rb) pathway and is enhanced by synergy with endocrine therapy. However, in TNBC, the lack of ER-driven signaling, higher genomic instability, and frequent alterations in the Rb pathway often limit the benefit of CDK4/6 inhibition as monotherapy, and resistance readily emerges through bypass mechanisms such as Cyclin E upregulation. In this context, PROTAC-based approaches—by eliminating CDK proteins rather than merely suppressing kinase activity—may achieve deeper pathway blockade and potentially overcome resistance mechanisms driven by protein overexpression or non-catalytic (scaffolding) functions, thereby providing a new avenue to explore CDK-targeted therapy in TNBC.

CDK4/6 promotes the G1/S transition by phosphorylating Rb, and CDK4/6 degradation suppresses Rb phosphorylation and induces cell-cycle arrest [137]. Palbociclib-based CDK4/6 degraders have shown inhibitory potential in TNBC preclinical models [56,58]; notably, a CRBN-recruiting palbociclib PROTAC achieved nanomolar degradation of CDK4/6 and inhibited Rb phosphorylation [58]. Beyond CRBN, a DDB1 and CUL4-associated factor 16 (DCAF16)-recruiting compound (A4) reduced CDK4/6 protein levels in a concentration- and time-dependent manner in MDA-MB-231 cells, showed lower cytotoxicity toward normal cells than palbociclib, and demonstrated in vivo therapeutic potential in xenograft models [56]. Together, these studies provide proof-of-concept that PROTACs can enable effective CDK4/6 targeting in TNBC.

Notably, IMiD-based CRBN ligands can induce degradation of neosubstrates such as Ikaros family zinc finger 1/3 (IKZF1/IKZF3), resulting in immunomodulatory effects [82,83]. Therefore, expanding to alternative E3 ligase recruitment strategies—such as VHL or IAP—may serve as potential routes to address CRBN loss-of-function resistance and may reduce certain neosubstrate-associated risks. Indeed, prior work has reported CDK6-selective degradation or dual CDK4/6 degradation with sustained antiproliferative effects [57]. From a resistance perspective, Cyclin E upregulation can bypass CDK4/6 dependence; accordingly, a bifunctional molecule (FN-POM) designed to degrade the Cyclin E–CDK2 complex showed synergistic growth inhibition with palbociclib in TNBC cell lines and organoids [138], supporting a combination concept that targets bypass nodes via degradation to overcome CDK4/6 inhibitor resistance.

The transcription-associated kinase CDK9 regulates RNA Pol II pause release and represents a potential vulnerability in tumors exhibiting “transcription-dependent survival” [139]. CDK9-selective inhibitor development is supported by an established medicinal chemistry foundation [140], and increased sensitivity has been observed in aggressive breast tumor models with high MYC expression [120]. In resistance-associated models, CDK9 upregulation has also been linked to enhanced MYC transcriptional elongation [141]. Moreover, CDK9 inhibition can downregulate short-lived pro-survival factors such as cellular FLICE-inhibitory protein (c-FLIP) and myeloid cell leukemia 1 (MCL1), thereby enhancing tumor necrosis factor-related apoptosis-inducing ligand (TRAIL)-induced apoptosis and providing a rationale for combination strategies [142]. Consistently, CDK9 degraders exhibit potent antiproliferative activity in vitro [15,59] and reduce downstream targets, including MYC [59]. However, because CDK9 is broadly expressed and essential in normal tissues, in vivo safety becomes a major bottleneck: despite strong in vitro degradation potency, animal studies may reveal a very narrow therapeutic window due to severe gastrointestinal toxicity [15]. This case exemplifies a central challenge in developing CDK-PROTACs for TNBC: for ubiquitously expressed, essential targets, potent antitumor activity achieved by degradation can coexist with substantial toxicity risk. Thus, restricting degrader activity to the tumor site becomes critical for translation. Prodrug design and exposure control (as well as delivery strategies) for such “high-potency but higher-risk” degraders are discussed in Section 6; for example, the TME-activated prodrug (P4) of the potent CDK4/6/9 degrader B5 achieved a balance between activity and safety in animal models [60].

In addition, multi-target degradation has been explored to increase network-level suppression. A celastrol-based PROTAC (compound 6a) selectively degraded glucose-regulated protein 94 (GRP94) and CDK1/4 in tumor cells and induced cell-cycle arrest and apoptosis while inhibiting tumor growth in the 4T1 model [143]; YX-112 degraded checkpoint kinase 1 (CHEK1) and phosphoinositide-3-kinase regulatory subunit 2 (PIK3R2) and more strongly suppressed CDK4 and phosphorylated AKT (p-AKT), suggesting a potential multi-axis mechanism involving DDR checkpoints, the PI3K/AKT pathway, and cell-cycle control [144]. Overall, these findings provide clear biological and PD support for CDK-focused degradation strategies in TNBC; nevertheless, the key translational issue remains how to maintain efficacy while controlling systemic exposure and toxicity (see Section 6) [15,60]. Key TNBC-relevant targets and representative degraders are summarized in Table 5.

### 5.4. Multifunctional Proteins Driving Invasion and Metastasis: Degradability of “Scaffolding Functions” in FAK/HDAC8/PTK6 and Related Targets

Death in patients with TNBC is largely attributable to invasion and metastasis, processes that are frequently driven by proteins with both catalytic and non-catalytic functions (e.g., scaffolding and protein–protein interaction modules). Conventional inhibitors typically block enzymatic activity only and often fail to abrogate scaffolding functions and interaction networks, creating a gap whereby pharmacological inhibition is weaker than genetic depletion with respect to migration/invasion phenotypes. By eliminating the target protein, PROTACs can simultaneously remove catalytic activity and scaffolding functions, providing a mechanistic advantage for anti-metastatic intervention. Below, representative examples—focal adhesion kinase (FAK), histone deacetylase 8 (HDAC8), and protein tyrosine kinase 6 (PTK6)—illustrate the shared rationale and practical value of PROTACs against such multifunctional proteins.

FAK exerts both kinase-dependent and scaffolding roles in migration, invasion, and survival. Its overexpression has been linked to altered signaling associated with tumor infiltration [148], and can promote survival and anoikis resistance through specific signaling axes, thereby supporting metastatic colonization [149]. Although FAK inhibitors have entered clinical investigation with acceptable tolerability [150], their coverage of scaffolding functions is limited. The FAK degrader PROTAC-3 efficiently downregulated FAK and more strongly suppressed migration and invasion in TNBC cells than a corresponding FAK inhibitor, exemplifying phenotypic gains of “degradation over inhibition” for multifunctional proteins [19].

A similar principle applies to HDAC8. The HDAC8 degrader CT-4 inhibited TNBC cell migration more effectively than its enzymatic inhibitor counterpart and achieved near-complete HDAC8 depletion in MDA-MB-231 cells, resulting in markedly enhanced anti-migratory activity. These findings suggest that, in TNBC, HDAC8 may function more prominently as a “migration/invasion phenotype target” than as a dominant proliferation driver [61].

For PTK6 (also known as breast tumor kinase, BRK), degradation further highlights the advantage of simultaneously intercepting multiple functional modalities. Because PTK6 has both kinase and non-kinase functions, inhibitors often fail to fully recapitulate loss-of-protein phenotypes. A VHL-recruiting PTK6 degrader reduced PTK6 abundance across multiple BC subtypes and triggered stronger apoptosis and antiproliferative effects, supporting the concept that degradation can cover non-catalytic functions and thereby influence both motility and survival programs [62].

When a target has broad physiological functions, selectivity becomes crucial. For example, heat shock protein 90 alpha (HSP90α) is a molecular chaperone for which pan-HSP90 inhibition/interference is often limited by systemic toxicity [63,145]. Although the HSP90 PROTAC BP3 showed antitumor activity in BC models, it was accompanied by body-weight loss, indicating that pan-HSP90 axis targeting may remain constrained by toxicity [145]. In contrast, selectively degrading HSP90α yielded improved antitumor efficacy with reduced toxicity in vivo, suggesting that isoform-selective degradation is a feasible approach to widen the therapeutic window [63]. In addition, degradation of histone methyltransferases such as euchromatic histone lysine methyltransferase 2 (EHMT2; G9a) and EHMT1 (GLP) reduced methylation marks and suppressed migratory phenotypes in BC cells, further indicating that certain epigenetic targets may be better positioned as “migration/metastasis-oriented” functional degradation targets [146].

Taken together, degradation of FAK, HDAC8, PTK6, and related targets supports a generalizable principle: PROTACs can more effectively suppress invasion/metastasis by concurrently removing catalytic and scaffolding functions. The HSP90α example further emphasizes that target-specific biology (e.g., broad physiological roles) necessitates precision strategies such as isoform-selective degradation to optimize the therapeutic window. Collectively, these studies provide degradation-based intervention concepts distinct from conventional inhibition for addressing TNBC invasion and metastasis.

### 5.5. Apoptosis and Tumor-Suppressive Networks: MDM2 Degradation and p53-Independent Vulnerabilities

In TNBC, tumor protein p53 (TP53) is among the most frequently mutated genes, and TP53 pathway inactivation occurs in the majority of cases, thereby attenuating apoptosis and genome stress responses and promoting therapeutic resistance [151]. MDM2 is the principal negative regulator of p53; however, in the context of p53 mutation or loss, conventional inhibitors that disrupt the MDM2–p53 interaction are ineffective. Emerging evidence indicates that direct degradation of MDM2 retains antitumor activity in p53-inactivated TNBC, suggesting that MDM2 may exert p53-independent oncogenic functions and/or serve as a critical network hub.

The MDM2-targeting PROTAC YX-02-030 suppressed the growth of p53-mutant TNBC both in vitro and in vivo by activating the p53 family member TAp73, thereby reinitiating apoptotic programs [64]. Its in vivo efficacy, together with the relatively weak activation of the p53 pathway in normal hematopoietic cells, further suggests that “degrading MDM2 itself”, compared with “inhibiting the MDM2–p53 interaction”, may confer a distinct safety profile [64]. This finding not only broadens the therapeutic scope of MDM2 targeting but also supports the concept that PROTACs can remodel protein–protein interaction networks and activate alternative tumor-suppressive pathways, thereby establishing actionable apoptosis triggers even when the canonical p53 axis is disabled. In addition to YX-02-030, natural product scaffold-derived MDM2 PROTACs have shown stronger antiproliferative and pro-apoptotic activities in p53-mutant TNBC models and exhibited tumor-suppressive signals in vivo, providing independent chemical validation for the conclusion that MDM2 degradation may be therapeutically relevant in p53-deficient TNBC [147].

Taken together, in receptor-poor TNBC, PROTAC technology demonstrates potential to expand the druggable landscape by enabling intervention against proteins that have been historically difficult to modulate. Nevertheless, TNBC heterogeneity and the broad physiological roles of certain targets can impose a narrow therapeutic window for PROTAC development [15]. Therefore, PROTAC research in TNBC should be tightly integrated with delivery technologies, prodrug strategies, and biomarker-driven patient stratification. Future advances will likely depend on a more precise understanding of TNBC subtype biology and on coupling potent degradation with smart systems that enable tumor-specific delivery and/or activation, thereby translating “high-potency degradation” into clinical benefit that is tolerable, stratifiable, and combinable.

## 6. Clinical Translation of PROTACs in BC: Current Evidence, Cross-Target Challenges, and Engineering Solutions

Although PROTAC technology—via its event-driven, catalytic degradation mechanism—has demonstrated substantial promise in preclinical and clinical research in BC, translation into safe, effective, broadly applicable therapeutics remains constrained by a series of biological and PK challenges. This section summarizes the available clinical evidence, systematically dissects the key bottlenecks, and highlights engineering-oriented strategy frameworks required to overcome these limitations—particularly those needed to broaden the therapeutic window in HER2-positive BC and TNBC.

### 6.1. Methodological Limitations and Areas of Contention

Across breast-cancer PROTAC programs, the major constraint on reliability is that most targets beyond ER remain supported primarily by in vitro datasets, with non-standardized endpoints (DC_50_/Dmax vs. IC_50_/GI_50_), variable exposure times, and potential confounding by hook effects and time-dependent degradation kinetics. Mechanistic attribution also varies: while best-practice studies include E3-inactive controls, proteasome-inhibition rescue, and orthogonal PD assays (e.g., immunoblot plus proteomics), other reports infer a “degradation-driven” benefit mainly from downstream phenotypes without fully excluding scaffold-related cytotoxicity or recruiter/E3-associated off-target liabilities (particularly for IMiD-based CRBN recruiters).

In vivo validation is often limited by small cohorts, restricted model diversity, and insufficient tumor PK–intratumoral PD–toxicity linkage, which is especially consequential in solid tumors where penetration and therapeutic window dominate translatability. Therefore, apparent “deep degradation” in vitro should not be assumed to translate into net clinical benefit unless supported by exposure-linked intratumoral PD and tolerability under clinically plausible dosing.

### 6.2. Clinical Evidence and Pipeline Landscape

Clinical translation of PROTACs in BC shows clear subtype-dependent heterogeneity. To date, the most mature clinical proof-of-concept evidence is concentrated in ER-positive/HER2-negative advanced BC, where an ERα PROTAC has advanced to and completed a randomized phase III trial. In contrast, degraders targeting vulnerabilities relevant to HER2-positive disease and TNBC largely remain in the preclinical or early clinical stage, and exposure, selectivity, and therapeutic window in the solid-tumor setting remain key limiting factors (see Section 6.3 and Section 6.4).

#### 6.2.1. VERITAC-2 (Vepdegestrant vs. Fulvestrant): Benefit in the ESR1-Mutant Subgroup and Safety Profile

Early-phase studies of vepdegestrant demonstrated manageable safety and pharmacodynamic evidence of ER degradation across evaluated dose levels and supported further clinical development [152]. In a Japanese phase 1 study, vepdegestrant 200 mg once daily was well tolerated and showed pharmacokinetic profiles comparable to those observed in Western patients, supporting global development and phase III implementation of the 200 mg once-daily regimen [153].

The phase III VERITAC-2 trial enrolled patients with ER-positive/HER2-negative advanced BC previously treated with CDK4/6 inhibitors plus endocrine therapy, and randomized them to vepdegestrant versus fulvestrant. A gatekeeping statistical strategy prioritized testing in the predefined ESR1-mutant subgroup and subsequently assessed consistency with the overall population [25]. The primary endpoint was PFS assessed by blinded independent central review (BICR) [25].

In the prespecified ESR1-mutant subgroup, vepdegestrant demonstrated a clearer improvement in PFS versus fulvestrant (median PFS: 5.0 months vs. 2.1 months; hazard ratio (HR), 0.58; *p* < 0.001). In the overall intent-to-treat (ITT) population, the PFS difference was smaller and did not reach statistical significance (3.8 months vs. 3.6 months; HR, 0.83; *p* = 0.07). These findings suggest that clinical benefit is more likely linked to resistance mechanisms and molecular stratification. However, the lack of significance in the ITT population indicates that positioning in an unselected population requires further support; subsequent studies should focus on biomarker-guided selection, dosing sequence, and combination/sequencing optimization (see Section 6.2.2) [25].

Overall tolerability of vepdegestrant was manageable; however, grade ≥3 adverse events occurred slightly more frequently than with fulvestrant (23.4% vs. 17.6%), while treatment discontinuation rates remained low (2.9% vs. 0.7%). Common adverse events included fatigue, elevations in transaminases, and nausea. Accordingly, baseline and periodic liver function monitoring (AST/ALT and bilirubin) and symptom-directed assessment (fatigue, nausea/diarrhea) are recommended, with prompt dose interruption/reduction for clinically meaningful abnormalities to prioritize safety [25].

Collectively, VERITAC-2 provides pivotal evidence that TPD can deliver clinical benefit in ER-positive BC in a biomarker-dependent manner; nevertheless, the magnitude of benefit in the overall population and long-term outcomes requires more mature OS data, deeper subgroup/biomarker analyses, and real-world evidence to define optimal clinical use [25].

The VERITAC-2 pattern—clear PFS benefit in ESR1-mutant disease but not in the ITT population—supports a mechanism-matched positioning: ER-PROTACs are more likely to deliver net benefit when endocrine resistance remains ER-dependent (e.g., ESR1 mutations), whereas ESR1–wild-type progression after CDK4/6 inhibitor therapy may be more bypass-driven, limiting incremental benefit from deeper ER lowering alone. From a safety standpoint, the manageable discontinuation rate and largely “oral targeted-therapy–like” toxicity profile support feasibility for chronic use, but future work should prioritize (i) sequencing after prior fulvestrant/SERDs (excluded in VERITAC-2) and (ii) combination tolerability with CDK4/6 or PI3K–AKT–mTOR inhibitors [25].

In addition, preliminary clinical signals for combination with CDK4/6 inhibitors have been reported [154]. These findings remain exploratory, and whether such combinations can broaden the benefiting population and/or increase the depth and durability of response will require confirmation in randomized controlled trials.

#### 6.2.2. Early Clinical Signals from Other ERα Degraders (e.g., AC699) and Preclinical Candidates

In parallel with the advancement of vepdegestrant to phase III evaluation, multiple next-generation ERα degraders are being developed using a broadly convergent strategy: preferential enrichment or stratification of populations with ESR1 mutations and/or endocrine resistance; oral administration; enhancement and/or prolongation of ER degradation; and exploration of combination or sequencing approaches with CDK4/6 inhibitors or agents targeting the PI3K–AKT–mTOR pathway, to increase depth of response and expanding the treatable population.

AC699 provides an illustrative example. Its first-in-human phase I study (NCT05654532) has thus far been disclosed mainly through conference abstracts, reporting antitumor activity signals in heavily pretreated patients with ER-positive/HER2-negative advanced BC (abstract-reported objective response rate (ORR): 33% in evaluable patients and 67% in the ESR1-mutant subgroup) [26]. Importantly, these data represent early findings from a small, non-randomized cohort with limited follow-up and are therefore best interpreted as supportive of continued development and biomarker-stratified validation, rather than as a basis for cross-trial comparisons versus standard therapies or other degraders. In particular, the current public disclosure provides limited safety granularity (e.g., grade ≥ 3 AE spectrum, dose interruptions/reductions, and treatment discontinuations), which constrains risk–benefit assessment for chronic oral use and for future combination strategies. Durability (DoR) and exposure-linked tolerability should be prioritized alongside ORR in subsequent updates to support clinical positioning. Accordingly, these early signals should be viewed as exploratory until confirmed with mature duration-of-response data and comparator-controlled designs. Key next steps include validating the predictive value of ESR1 mutations in larger cohorts, defining the relationship between PK/PD (magnitude and duration of ER degradation) and clinical endpoints, and assessing the incremental benefit of combination regimens under more rigorous study designs.

At the preclinical level, candidates such as UM-ERD-3111/4001 and HP568 indicate continued rapid optimization in this space. AACR abstracts have reported that UM-ERD-3111 and UM-ERD-4001 exhibit subnanomolar ER degradation activity with favorable oral properties, reduce both wild-type and mutant ER protein levels in vivo, and generate PD and antitumor signals, including tumor regressions [155]. For HP568, an abstract highlighted potent degradation of wild-type ER and multiple ESR1 mutants, and suggested potential synergy when combined with CDK4/6 inhibitors [156]. Because these reports are largely conference abstracts, methodological details and reproducibility are limited; accordingly, claims implying superiority over existing molecules or head-to-head advantages should be treated conservatively in a review and presented only as hypothesis-generating. Ultimately, clinical translatability will depend on whether human exposure can consistently reach levels required for effective degradation, the safety profile under long-term dosing (including potential CRBN-related liabilities), and whether the association between biomarkers (e.g., ER dependency state and ESR1 mutation status) and clinical benefit remains consistent across heterogeneous patient populations.

Overall, apart from vepdegestrant, most ERα degraders remain in early-stage development. At present, a clearer research priority is to use resistance-associated biomarkers such as ESR1 mutations as the primary validation setting and to improve depth and breadth of benefit through combination and/or sequencing strategies (see Section 6.2.2) [25,30].

### 6.3. Cross-Subtype Shared Challenges: Resistance, Biomarkers, and the Verifiability of Combination Strategies

#### 6.3.1. E3-Axis/Ternary-Complex-Driven Acquired Resistance

With prolonged PROTAC treatment, acquired resistance may emerge through mechanisms that differ from those observed with conventional inhibitors; these mechanisms frequently involve multiple interconnected layers, including the “E3–ternary complex–intracellular exposure” axis [34]. On the E3 side, tumor cells may downregulate or mutate CRBN, VHL, or other E3 ligases and/or their complex components, thereby reducing PROTAC recruitment and weakening productive degradation. On the POI side, mutations at the binding interface, conformational alterations, or compensatory upregulation of POI expression can all diminish the formation and/or stability of the ternary complex. On the exposure side, decreased drug uptake, increased activity of efflux transporters (e.g., P-glycoprotein (P-gp)), and insufficient penetration into solid tumors can lower the effective intracellular concentration. In addition, altered cellular uptake/efflux capacity and adaptive changes in the UPS may further reduce effective degradation [34].

Accordingly, key directions for sustaining long-term efficacy with next-generation PROTACs include developing alternative degraders that can switch E3 ligases, exploring multi-E3-compatible or multivalent designs, and implementing rational combinations with other therapies to reduce selective pressure for resistance. Collectively, these mechanisms indicate that future resistance-mitigation strategies must extend beyond the target itself and adopt multidimensional interventions.

#### 6.3.2. Predictive and Dynamic Monitoring Biomarkers: From ESR1 Mutations to the E3 Ligase Landscape and PD Readouts

The clinical outcome of vepdegestrant in the ESR1-mutant subgroup suggests that predictive biomarkers can directly influence the identification of patients most likely to benefit from PROTAC therapy. For PROTACs targeting BET proteins, CDKs, or kinases, an implementable biomarker framework remains to be established for patient selection, response prediction, and resistance monitoring. Potential directions include the molecular status of the POI (e.g., mutations and gene fusions), intratumoral expression levels of key E3, molecular contexts that modulate the efficiency of ternary complex formation, and proteasome function–related signals that can be dynamically tracked through liquid biopsy [66]. Building a multidimensional biomarker system is therefore foundational for precision patient stratification, efficacy prediction, and longitudinal resistance surveillance.

#### 6.3.3. Combination and Sequencing Strategies: Mechanistic Synergy, Overlapping Toxicities, and Temporal Optimization

Combining PROTACs with established therapies is mechanistically well justified; however, clinical translatability depends on whether (i) synergy is verifiable, (ii) overlapping toxicities are manageable, and (iii) treatment timing and sequencing can be optimized. Beyond the clinical evaluation of ER-targeting PROTACs combined with CDK4/6 inhibitors, preclinical studies have proposed multiple combination paradigms. For example, BET degraders combined with immune checkpoint inhibitors may modulate the immunosuppressive microenvironment by downregulating molecules such as programmed death-ligand 1 (PD-L1) [65]. PARP1 degraders combined with ataxia-telangiectasia mutated (ATM) or ATM and Rad3-related (ATR) inhibitors may enhance synthetic lethality. Degradation of downstream kinase nodes (e.g., phosphoinositide 3-kinase p110α (PI3K-p110α)) combined with upstream pathway inhibitors may mitigate feedback reactivation and delay resistance [16]. Future clinical investigations should be grounded in deep mechanistic understanding and systematically evaluate the safety, efficacy, and optimal sequencing of these combination strategies.

#### 6.3.4. Safety, Toxicity, and Monitoring

PROTAC toxicities in BC should be anticipated from: (i) on-target/off-tumor depletion when the POI is physiologically important or broadly expressed; (ii) E3-recruiter–related liabilities (notably CRBN/IMiD recruiters with potential neosubstrate effects); (iii) PK/distribution limitations in solid tumors; and (iv) additive toxicities in combination regimens [24,81]. Clinically, a pragmatic minimum monitoring set includes baseline and periodic CBC and comprehensive metabolic panel with AST/ALT/bilirubin plus focused GI/fatigue surveillance, with heightened vigilance for degraders of essential transcriptional nodes (e.g., CDK9) given preclinical dose-limiting GI toxicity signals [15,25]. Management follows standard targeted-therapy practice (supportive care, hold/reduce, cautious re-challenge), but empiric dose escalation should be avoided unless pharmacodynamic engagement is evident because dose–degradation can be non-linear (hook effect) [76]. When systemic tolerability limits dosing, tumor-selective activation/delivery (prodrugs, antibody/aptamer-guided delivery, local administration) should be considered safety-enabling strategies [60,157].

#### 6.3.5. Patient-Centered Considerations (Quality of Life, Mental Well-Being, and Long-Term Outcomes)

Beyond mechanistic synergy and overlapping toxicities (Section 6.2.1), PROTAC translation should be evaluated with patient-relevant outcomes, since metastatic BC is often treated long term. QoL and treatment burden may change with administration logistics: oral ER-targeting PROTACs could reduce clinic visits and injection-site discomfort versus intramuscular fulvestrant, but may increase adherence variability, drug–drug interactions, and cumulative pill burden in combinations; thus, trials should embed validated PROs (e.g., EORTC QLQ-C30/BR23 or FACT-B) and time-to-deterioration endpoints. Mental well-being also warrants attention when biomarker-driven adaptation is used (e.g., serial ctDNA ESR1 monitoring): frequent “molecular surveillance” can induce anxiety and decisional stress, supporting routine counseling, shared decision-making, and psychosocial support [158]. Finally, long-term outcomes should be prospectively tracked because sustained depletion may yield cumulative effects; studies should follow fatigue trajectories and key survivorship domains (endocrine/metabolic symptoms, sexual health, bone/cardiovascular health) during prolonged exposure [159], and address reproductive safety for IMiD/CRBN-based recruiters in patients of childbearing potential [81].

### 6.4. Druggability Bottlenecks in Solid Tumors: Constraints Among Effective Exposure, Selectivity, and the Therapeutic Window

The intrinsic physicochemical properties of PROTACs pose clear translational barriers in the context of solid tumors. Typical PROTACs have molecular weights in the range of ~700–1000 Da and frequently fall outside Lipinski’s Rule of Five (Ro5), resulting in limited aqueous solubility and membrane permeability as well as suboptimal oral bioavailability [32,88]. Their large size and pronounced amphiphilicity also increase the likelihood of being substrates of efflux transporters such as P-gp, leading to active extrusion and reduced intracellular effective concentrations [33]. Moreover, conventional PROTACs generally lack active tumor-targeting capability; consequently, in vivo distribution is often non-specific, making it difficult to achieve and maintain sufficient effective exposure within tumor tissue—particularly in the poorly perfused, stroma-dense interior of solid tumors [45].

In addition, although “catalytic degradation” can enhance potency, under conditions of systemic non-specific distribution, it may also amplify on-target risks in normal tissues. Because PROTACs rely on E3 (e.g., CRBN and VHL) and the proteasome system, both broadly expressed across tissues, “on-target, off-tumor” toxicities may arise when the POI is also expressed in normal tissues. This concern is particularly prominent when the target is ubiquitously expressed or essential for cell survival (e.g., CDK9), in which case the therapeutic window may be extremely narrow and may even exhibit an “inverse therapeutic index” [15].

Accordingly, in solid-tumor subtypes such as HER2-positive disease and TNBC, PROTAC development is often constrained by the mutual trade-offs among potency, intratumoral effective exposure, and systemic safety. In the absence of tumor-selective delivery or selective activation strategies, these three parameters are difficult to optimize simultaneously, which may partly explain the relatively slow progress of PROTACs directly targeting HER2 or broadly expressed kinases in solid tumors [45]. Overcoming this bottleneck typically requires not only chemical optimization of the degrader itself but also external strategies—such as delivery approaches and exposure control—to improve tumor selectivity and expand the therapeutic window.

### 6.5. Engineered Translational Strategies: Delivery, Conditional Activation, Cell Selectivity, and Local Permeabilization to Expand the Therapeutic Window

**Figure 4:**
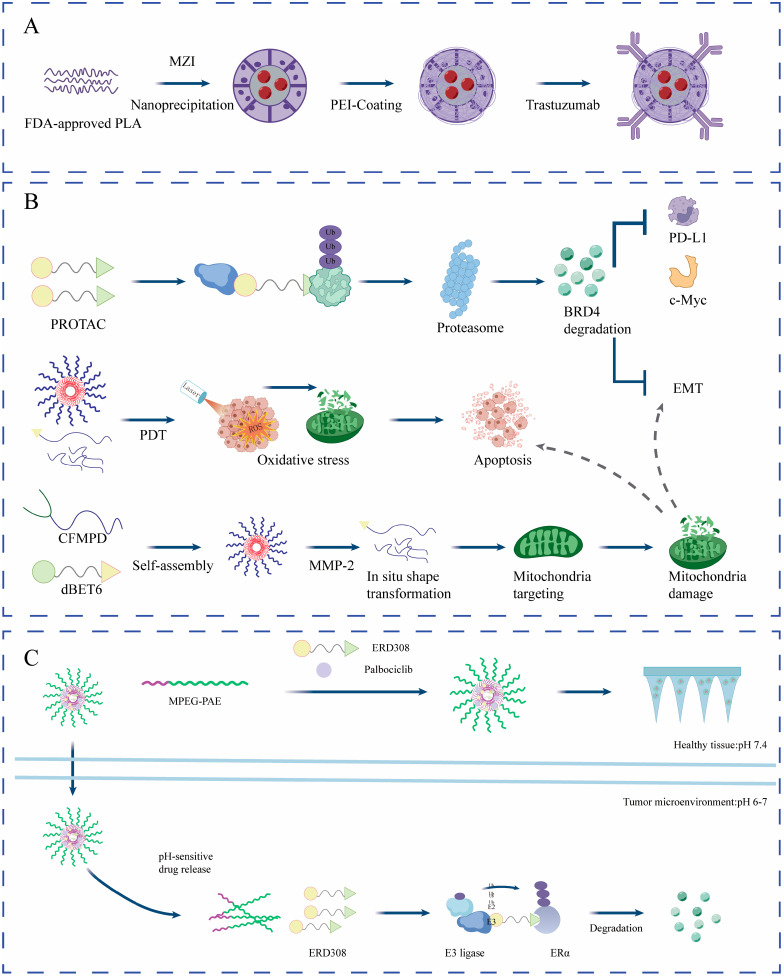
Representative engineering strategies for expanding the therapeutic win-dow of PROTACs in BC via engineered delivery and local administration. (**A**) Trastuzumab-modified polymeric nanoparticles (MZ1-ACNPs) enhance the cellular uptake and cytotoxicity of the BRD4 degrader MZ1 in HER2-positive tumor cells through HER2-mediated binding and endocytosis, thereby improving the balance between effective exposure and selectivity [37]. (**B**) A matrix metalloproteinase-2 (MMP-2)-responsive, dBET6@CFMPD enables deep tumor penetration and controlled release upon activation in the TME. By combining BRD4 degradation (dBET6) with PDT, this platform induces tumor growth inhibition, suppresses metastasis, and elicits PD changes related to the immune microenvironment [169]. (**C**) A hyaluronic acid microneedle patch integrating pH-responsive micelles enables local co-delivery of the ER degrader ERD-308 and palbociclib, achieving sustained local release and synergistic antitumor activity while improving the safety window by reducing systemic exposure [167].

To mitigate the “exposure–selectivity–safety window” trade-off discussed in Section 6.4, actionable engineering-based countermeasures can be broadly categorized into four approaches: (1) delivery systems to increase intratumoral exposure and tissue penetration; (2) conditional activation/stimulus-responsive designs to reduce off-tumor degradation; (3) cell-type-specific delivery to confine degradation events to antigen-positive cells; and (4) local administration and physical permeabilization strategies to trade reduced systemic exposure for enhanced local pharmacological activity in accessible lesions. Representative approaches are summarized in Figure 4 and Table 6.

**Table 6:** PROTAC Engineering Strategies: From Delivery to Conditional Activa-tion.

Strategy Category	Specific Subcategory	Core Mechanism	Representative Studies/Systems
Delivery Systems: Enhancing Tumor Exposure and Penetration	Liposomes/Lipid Nanoparticles	Improved PK and tumor deposition: Encapsulate hydrophobic PROTACs, enhance solubility, prolong circulation time; achieve passive/active targeting via EPR effect or surface modification Therapeutic window remodeling: e.g., improved toxicity profile of CDK9 PROTAC (THAL-SNS-032)	Liposomes co-delivering BRD4 degrader ARV-825 and docetaxel (synergistic efficacy with immune microenvironment modulation) [65] Liposomes co-loading photosensitizer and BRD4 degrader MZ1 (L@NBMZ), enabling synergy between degradation and PDT [160]
	Polymeric Nanoparticles/Micelles	Multi-responsive design and deep penetration: Integrate responsive elements (MMP-2, pH, GSH) for TME-triggered drug release and deep tumor penetration Multi-stage response design: MMP-2 cleavage enables deep penetration, followed by rapid pH/GSH-triggered release after cellular entry, addressing penetration and intracellular release barriers	MMP-2/pH/GSH multi-responsive polymer-PROTAC micelles achieving deep tumor penetration and intracellular specific release [45] Trastuzumab-modified polymeric nanoparticles (MZ1-ACNPs) for active targeted delivery in HER2+ BC [37]
Conditional Activation/Stimuli-Responsive	Tumor Microenvironment-Responsive Prodrugs	Reducing “on-target, off-tumor” toxicity: PROTAC remains inactive or minimally active in systemic circulation, selectively activated within tumor-specific biochemical environment (high GSH, high ROS, low pH, hypoxia) Leveraging metabolic differences: GSH concentration gradient (100–1000 fold) between tumor and normal tissues provides a basis for selective activation	GSH-responsive ERα PROTAC prodrug activated in tumor reductive environment [161] ROS-responsive prodrug [162] Hypoxia-responsive strategy (concept) [163]
	Exogenous Stimuli-Triggered	Achieving high spatiotemporal precision: Utilize exogenous stimuli (light, radiation) for site-specific activation at defined times, minimizing systemic exposure	Light-controlled PROTAC (TMP-TAC-PC) enabling light-dependent degradation regulation of engineered protein tag (eDHFR) [164] X-ray-responsive PROTAC nanomicelles (RCNprotac) with radiotherapy-triggered release and radiosensitization effects [165] Gold nanocube co-delivery system (AuPLs) for photothermal-triggered bioorthogonal generation of active PROTAC [166]
Cell Type-Specific Delivery	Antibody/Aptamer-PROTAC Conjugates	Fundamentally restricting degradation events to specific cell populations: Utilize tumor cell surface-specific or overexpressed antigens (e.g., HER2, NCL) as “navigation anchors” to achieve ligand-receptor mediated endocytosis and intracellular release Core value: Elevating “selectivity” from molecular binding to the cellular recognition level Engineering challenge: Endosomal escape is the key rate-limiting step; it requires optimized linker design or combination with endosome-disrupting agents to enhance cytosolic delivery efficiency [41,42,43]	Antibody-PROTAC conjugate (Ab-PROTAC): Trastuzumab-Ab-PROTAC3 achieving BRD4 selective degradation in HER2-positive cells [36] Aptamer-PROTAC: AS1411 aptamer-ZL216 targeting surface nucleolin (NCL) for selective degradation [38]
Local Delivery and Physical Permeabilization	Microneedles/Local Patches	Trading systemic exposure for local efficacy: Direct delivery of PROTACs to local lesions (e.g., subcutaneous tumors, superficial metastases), achieving high local concentration and sustained release while substantially reducing systemic exposure and toxicity	Hyaluronic acid microneedle patch: Co-delivery of ER degrader ERD-308 and palbociclib, achieving local sustained release, synergistic tumor inhibition, and reduced systemic exposure [167]
	Ultrasound-Targeted Microbubble Destruction	Physical enhancement of local penetration and accumulation: Utilize ultrasound cavitation effects to transiently increase local vascular and tissue permeability, promoting intratumoral drug enrichment and penetration	Ultrasound-enhanced PROTAC tumor penetration: ARV-825 loaded microbubbles combined with tumor-directed ultrasound to enhance local release and uptake [168]

#### 6.5.1. Exposure-Enhancing Delivery: Nanoparticle/Lipid/Polymer Platforms to Improve Tumor Deposition and Penetration

Liposomes and lipid nanoparticles are frequently employed to improve the in vivo exposure profiles of PROTACs and, to some extent, enhance tumor deposition. Using the CDK9 degrader THAL-SNS-032 as an example, lipid-based nanoparticle delivery can sustain target degradation and antiproliferative activity across multiple BC cellular models, while partially improving the toxicity profile in normal cells. These findings suggest that formulation engineering may broaden the therapeutic window [92]. In combination-oriented designs, liposomal co-delivery of the BRD4 degrader ARV-825 and docetaxel demonstrated stronger tumor-suppressive activity in BC models, accompanied by immune-related changes such as PD-L1 downregulation following BRD4 degradation. This provides mechanistic clues supporting a coordinated “degrader–chemotherapy–immunity” strategy [65]. In addition, co-encapsulation of a photosensitizer with the BRD4 degrader MZ1 in liposomes (L@NBMZ) enables the integration of protein degradation with photo-induced cell death, highlighting the ability of delivery platforms to combine multiple mechanisms of action [160].

Polymeric nanoparticles and micellar systems more readily accommodate active targeting and multi-stage, triggerable drug release. Trastuzumab-modified polymeric nanoparticles delivering the BRD4 degrader MZ1 (MZ1-ACNPs) enhance uptake and cytotoxicity in HER2-positive cells via antibody-mediated endocytosis, indicating that “antigen-guided navigation plus encapsulated delivery” may simultaneously improve selectivity and exposure in HER2-positive BC [37]. Multi-responsive polymer–PROTAC micelles can be engineered to improve deep tumor penetration through cleavage by matrix metalloproteinase 2 (MMP2), and subsequently disassemble under intracellular acidic conditions and high glutathione (GSH) levels to release the payload. Such designs aim to address limited penetration in solid tumors and suboptimal intracellular drug release [45]. Notably, whether a delivery system can translate into clinical benefit ultimately depends on establishing a robust exposure–PD–efficacy relationship in clinically relevant models—specifically, a stable linkage among intratumoral exposure, the magnitude/duration of POI degradation, and therapeutic outcomes—as well as the feasibility of scalable manufacturing and reliable batch-to-batch consistency control.

#### 6.5.2. Conditional Activation: TME-Responsive and Externally Triggered Strategies to Reduce Off-Tumor Degradation

When systemic distribution results in TPD in non-tumor tissues and causes dose-limiting toxicities (DLTs), simply increasing intratumoral exposure may not necessarily broaden the therapeutic window. A more direct strategy is to engineer PROTACs to remain minimally active in circulation and to be selectively activated within the TME or inside tumor cells. For example, GSH-responsive prodrugs can reversibly mask key functional groups of ERα PROTACs, thereby enabling preferential activation in the reductive milieu of tumor cells and reducing the risk of off-tumor degradation [161]. Hypoxia-responsive, ROS-responsive, and radiotherapy (X-ray)-triggered PROTACs further provide spatiotemporally constrained activation paradigms by enabling in situ activation within tumor regions [162,163,165]. In nanomedicine-enabled designs, an MMP2-responsive, shape-transformable, and mitochondria-targeted formulation (dBET6@CFMPD) couples BRD4 degradation with photodynamic therapy (PDT), enhancing tumor growth suppression and metastatic control in BC models while producing immune-related PD outputs. Collectively, these results underscore the integrated value of “delivery–controlled release–synergistic therapy” [169].

Exogenous triggers can further improve the spatiotemporal precision of degradation. A light-controllable trimethoprim (TMP)-based targeting chimera (TMP-TAC) system, which uses an Escherichia coli dihydrofolate reductase (eDHFR) tag as the recognition module, enables optical control of degradation through either photocaged or photoswitchable designs. In particular, TMP-TAC-PC enhances degradation of eDHFR-fusion proteins upon irradiation at ~375–405 nm and can be applied to modulate chimeric antigen receptor (CAR)–eDHFR surface expression as well as downstream cytotoxicity/cytokine release, supporting the feasibility of using light as a “TPD on–off switch” [164]. However, this system relies on engineered tags and short-wavelength light, and translation to deep-seated solid tumors will likely require red-shifted activation, delivery support, and/or localized irradiation strategies.

An X-ray-responsive PROTAC nanomicelle platform (RCNprotac) leverages radiotherapy as a release signal; BRD4 degradation subsequently suppresses DNA damage repair and produces radiosensitization, providing a delivery-based implementation route for “radiotherapy–degrader” combination strategies [165]. In addition, a gold nanocube–based PROTAC co-delivery nanoplatform (AuPLs) uses photothermal heating to melt phase-change materials, releasing a PROTAC prodrug and a catalyst and enabling in situ generation of the active degrader via a bioorthogonal reaction. This further illustrates the feasibility of stepwise control over the activation process [166]. For clinical translation, key questions remain, including the robustness of activation thresholds across heterogeneous TMEs, the accessibility and dosimetric boundaries of exogenous triggering in deep lesions, and the safety implications of post-activation drug redistribution.

#### 6.5.3. Cell-Selective Delivery: Antibody-/Aptamer-PROTAC Conjugates to Confine Degradation to Antigen-Positive Cells

For tumors with surface antigens that are tumor-specific or highly overexpressed (e.g., HER2-positive BC), PROTACs can be conjugated to targeting moieties such as antibodies or aptamers. Following antigen-mediated endocytosis, the active payload is released intracellularly, thereby restricting the degradation event to antigen-positive cells.

For instance, Ab-PROTAC3, a trastuzumab-conjugated BRD4 PROTAC, enables a “HER2-mediated internalization–intracellular enzymatic cleavage–BRD4 degradation” cascade and induces apoptosis in HER2-positive cells [36]. In another example, ZL216, a PROTAC guided by the NCL aptamer AS1411, achieves selective NCL degradation and antiproliferative activity in BC cells [38]. Although this strategy is conceptually extendable to other antigens such as trophoblast cell surface antigen 2 (Trop-2) and EGFR, in vivo efficacy is jointly constrained by multiple factors, including circulatory stability of the conjugate, internalization efficiency, and intracellular release kinetics, antigen-expression heterogeneity, and potential immunogenicity. Continued optimization of these key parameters is therefore required.

#### 6.5.4. Local Administration and Physical Permeabilization: Trading Reduced Systemic Exposure for a Local PK/PD Closed Loop

In settings such as superficial lesions, locoregional recurrence, or disease stages with an interventional window, local delivery can reduce systemic exposure to mitigate systemic toxicities while achieving higher effective concentrations at the lesion site. A microneedle patch co-loading the ER degrader ERD-308 and palbociclib into pH-responsive micelles and integrating them into a hyaluronic acid microneedle system enabled local sustained release and tumor inhibition in BC models, while alleviating systemic exposure burden [167]. Ultrasound-targeted microbubble destruction (UTMD) can transiently increase local permeability via acoustic cavitation; ARV-825–loaded microbubbles combined with ultrasound irradiation at the tumor site promoted local release and uptake, thereby enhancing degradation and antitumor activity [168].

Because local delivery is more likely to apply to specific lesion types and treatment stages, future studies should more clearly define indication boundaries, feasibility of repeated administration, local tissue reactions, and the strength of association between local PD changes and long-term outcomes, to avoid a disconnect between “local activity” and “clinical benefit”. A key advantage of this approach is that it more readily enables a verifiable “local exposure–extent of degradation–efficacy” closed loop, providing more quantitative support for dose selection and safety management, particularly when local PK can be directly characterized [167].

## 7. Conclusions and Perspectives: From Mechanistic Advantages to Clinically Implementable Strategies That Are “Stratifiable, Deliverable, and Manufacturable”

PROTACs provide an additional mechanistic dimension for targeted therapy in BC, shifting the paradigm from “continuous occupancy-based inhibition” to “event-driven elimination”. By reducing the abundance of pathogenic proteins, PROTACs may simultaneously suppress both the catalytic activity and non-catalytic/scaffolding functions of the POI and, in selected resistance contexts, achieve more durable pathway suppression. However, clinical benefit does not necessarily scale linearly with the magnitude of degradation observed in vitro. Translational feasibility instead depends more critically on the level of evidence within each molecular subtype, biomarker-driven patient stratification, and—particularly in solid tumors—whether an acceptable therapeutic window can be established together with a verifiable PK/PD–efficacy linkage.

Current evidence indicates marked “subtype-dependent divergence” in the translational trajectory of PROTACs in BC. In ER-positive BC, the evidence supporting ERα degraders is the most mature. For example, early studies of vepdegestrant have defined feasible dosing and an initial safety profile [153]. In the phase III VERITAC-2 trial, PFS improvement in the overall population did not reach statistical significance; however, a clearer PFS benefit signal was observed in the prespecified ESR1 mutation subgroup [25]. Collectively, these findings suggest that: (1) TPD is not intrinsically “superior” and may rely on matching specific resistance mechanisms with appropriate biomarker-based stratification; and (2) dynamically monitorable biomarkers such as ESR1 mutations provide an actionable framework for treatment timing and switching decisions. Meanwhile, early clinical data for oral ERα degraders such as AC699 have also suggested stronger activity signals in patients with ESR1 mutations, supporting further validation of true clinical benefit and the optimal treatment sequence (NCT05654532) [26].

In contrast, in solid-tumor settings such as HER2-positive BC and TNBC, the key challenge is often not “whether the target can be degraded”, but rather “whether sufficient intratumoral exposure can be achieved without compromising safety”. Factors including high molecular weight, limited tissue penetration, enhanced efflux transport, and non-specific in vivo distribution may readily lead to the coexistence of “insufficient tumor exposure” and “systemic exposure–related toxicities”, thereby exacerbating the trade-off among effective exposure, tumor selectivity, and the therapeutic window. For targets that are broadly expressed and biologically essential (e.g., CDK9), even highly potent in vitro degradation may translate into a very narrow therapeutic window due to on-target/off-tumor toxicity [15].

Therefore, a more translationally meaningful direction is to position PROTACs as high-efficiency TPD payloads and, through engineering strategies, confine the degradation event as much as possible to tumor cells or tumor regions. Representative approaches include Ab-PROTACs (e.g., a framework in which HER2-mediated endocytosis enables intracellular PROTAC release [36]), nanodelivery to enhance tumor deposition and deep penetration [45], and prodrug/stimulus-responsive designs that enable conditional activation in response to the TME or exogenous triggers (e.g., GSH-, hypoxia-, ROS-, or X-ray-triggered activation [161,164,165]). The overarching goal of these strategies is to establish interpretable and verifiable intratumoral PK/PD linkages, thereby providing a rational basis for dose selection and safety management.

Looking ahead, the next-stage breakthroughs for PROTACs in the BC field can be summarized into four interrelated directions:

(1) Expansion of E3 and switchable recruitment

Current development relies heavily on CRBN and VHL, which increases the risk of resistance and limits tissue selectivity. There is a need to expand the ligand repertoire for E3 and, together with evaluations of their expression and functional availability in BC-relevant tissues, develop recruitment strategies with stronger tissue preference and more controllable risk profiles. In parallel, “switchable E3” designs or multi-E3–compatible architectures should be advanced to reduce selective pressure imposed by reliance on a single degradation pathway [88].

(2) Establishing a reproducible “intratumoral PK/PD–efficacy/toxicity” closed loop

In solid tumors, verifiability is often more critical than the in vitro degradation concentration 50% (DC_50_). In models that more closely reflect patients—such as PDXs, organoids, and immunocompetent models—efforts to capture patient-specific heterogeneity have advanced, with patient-derived organoids offering particular advantages for translational interrogation. However, key limitations remain in standardization, reproducibility, scalability, and the lack of fully integrated immune and vascular components [8]. Given these limitations, several key questions must be addressed to enable robust translation: whether true intratumoral exposure reaches a required threshold; how to quantify the magnitude and duration of degradation; and whether these parameters can be linked to efficacy and toxicity through relationships that are transferable across models and settings. Without such a closed loop, delivery and prodrug strategies are unlikely to progress into clinically scalable development with robust validation.

(3) Biomarker-driven precision use and rational combinations

The VERITAC-2 findings in the ESR1 mutation subgroup underscore the importance of predictive biomarkers [25]. Going forward, more actionable stratification frameworks are needed for BET proteins, CDKs, kinases, and other targets. Beyond target alterations, stratification should incorporate additional dimensions such as E3 expression profiles, factors governing ternary-complex formation, proteasome activity, and TME characteristics [66]. On this basis, mechanistically coherent combination and sequencing strategies should be developed—such as ERα degraders plus CDK4/6 inhibitors, BET degradation plus immunotherapy, and degradation of DDR nodes plus synthetic-lethal combinations—while optimizing scheduling and dosing to mitigate overlapping toxicities.

(4) Manufacturing and quality control, accessibility, and long-term safety assessment

Given their structural complexity, PROTACs pose challenges in synthesis and quality control. Delivery/conjugation systems further introduce hurdles in chemistry, manufacturing, and controls (CMC), scale-up, batch-to-batch consistency, and cost. Moreover, long-term and deep depletion of a POI may entail chronic safety risks distinct from those of conventional inhibitors, necessitating more systematic preclinical toxicology and long-term follow-up to define safety boundaries—particularly when the target has essential physiological roles in the cardiovascular, skeletal, or reproductive systems.

Importantly, PROTAC development should be judged not only by biomarker-stratified efficacy and therapeutic window, but also by patient-centered endpoints—QoL, mental well-being under monitoring, and long-term functional tolerability—to ensure sustainable real-world care [159].

Taken together, the development of PROTACs in BC is transitioning from “proof-of-concept” toward clinically implementable strategies that are “stratifiable, deliverable, and manufacturable”. ER-positive BC has already demonstrated the clinical feasibility of TPD under biomarker-based molecular stratification [25]. By contrast, solid-tumor settings such as HER2-positive disease and TNBC more clearly indicate that engineered delivery and conditional activation are often prerequisites for establishing a therapeutic window and advancing clinical development. With continued expansion of E3, maturation of engineering strategies, and refinement of biomarker-driven trial designs, PROTACs are expected to define more predictable and reproducible beneficiary populations across BC subtypes and to integrate with existing treatment paradigms through more rational combination and sequencing approaches.

## Figures and Tables

**Table 1:** Comparison of PROTAC Strategies Across BC Molecular Subtypes.

Subtype	Current Treatment Framework	Major Unmet Needs/Bottlenecks (Mechanistic–Clinical)	PROTAC Entry-Point Rationale	PROTAC R&D Directions (Targets and Modalities)	Translational Considerations & Stratification Variables
Hormone receptor–positive/HER2-negative	Endocrine therapy (AI/SERM/SERD) as backbone, ± CDK4/6 inhibitors [3,4] Selected populations: PI3K/AKT/mTOR pathway-targeted agents [14]	ESR1 LBD mutations (Y537S, D538G) drive ligand-independent activation and resistance [12,13] Fulvestrant: intramuscular administration and exposure limitations, which may contribute to incomplete ER downregulation in some clinical contexts [11,22]. Disease progression after resistance, OS plateau [10,11]	Event-driven clearance: Direct reduction of ERα protein abundance counteracts mutation-driven conformational activation [23] Catalytic and deep clearance: More active and programmable degradation compared to SERDs, aiming for deeper and more durable pathway suppression [24]	Clinical lead: ARV-471 [25] Early clinical: AC699 [26] Preclinical new molecules: UM-ERD-3111/4001, HP568 Mechanistic expansion: Alternative interface: ERE-PROTAC (targets DBD, bypassing LBD mutations) [27] Coactivator targeting: SRC-3 PROTAC [28] Dual targeting: ERα/aromatase dual-degradation PROTAC [29]	Core stratification: ESR1 mutation status (ctDNA dynamic monitoring) [30] Combination strategies: With CDK4/6 inhibitors, PI3Kα/mTOR inhibitors [31] Key challenges: Exposure and delivery: Oral bioavailability, tumor penetration [32,33] Resistance: E3 ligase (e.g., CRBN) downregulation/mutation, altered ternary complex stability [34]
HER2+	Anti-HER2 monoclonal antibodies, TKIs, ADCs as core therapies [6,15] Limited options post-resistance, poor prognosis [16]	Heterogeneous resistance mechanisms: HER2 mutations, compensatory activation of downstream PI3K/AKT/mTOR pathway, kinome reprogramming [16] HER2 expressed in normal tissues; systemic potent inhibition/degradation risks "on-target, off-tumor" toxicity [15]	“Delivery-driven” logic: Leveraging HER2 overexpression as a tumor-selective delivery anchor, rather than merely a degradation target Resensitization strategy: Degrading key downstream resistance nodes (e.g., PI3K-p110α) to restore sensitivity to prior therapie [16]	Direct degradation: Lapatinib-derived HER2 PROTAC [35] Targeted delivery systems: Ab-PROTAC: Trastuzumab-PROTAC conjugates [36] Antibody-modified nanoparticles: Trastuzumab-ACNPs delivering MZ1 [37] Aptamer-PROTAC: Targeting surface nucleolin (NCL) [38] Downstream node degradation: PI3K-p110α PROTAC [16]	Stratification and selection: HER2 expression level [39,40], endocytosis efficiency and intracellular release kinetics [41,42,43], downstream pathway activation status [16,44] Development key: PK/PD of conjugates/nanosystems, tumor-specific distribution, intracellular release efficiency [45] Therapeutic window management: Avoiding normal tissue toxicity from systemic exposure [15]
TNBC	Chemotherapy as backbone, but resistance common and toxicity substantial [17,46] Immune checkpoint inhibitors for PD-L1+ populations [17]	Lack of definitive targets, high heterogeneity [4,47] Reliance on largely "undruggable" targets (transcription factors, scaffolding proteins) [9] High invasive and metastatic capacity, poor prognosis [4,48]	Expanding target landscape: Eliminating proteins refractory to conventional inhibition (transcriptional/epigenetic regulators, scaffolding proteins) [20,21] Network remodeling: Achieving deep phenotypic suppression (proliferation, metastasis, DNA repair) by degrading key hubs [19]	Transcriptional/Epigenetic regulation: BET/BRD4: ARV-825, BETd-246 (degradation superior to inhibition) [49,50] PRMT5: Novel CRBN-recruiting degrader [51] c-Myc: Aptamer/oligonucleotide-mediated PROTAC-like strategies [52] DNA damage repair: PARP1 PROTAC: NN3 (induces ferroptosis, overcomes certain resistance) [53] CDK12/13 PROTAC: Synergy with PARPi (synthetic lethality) [54,55] Cell cycle/Kinases: CDK4/6 PROTAC: Palbociclib-based, utilizing different E3s (CRBN/VHL/DCAF16) [56,57,58] CDK9 PROTAC: Potent but systemically toxic, requires prodrug strategies (e.g., B5 modified to TME-activated prodrug P4 achieving balanced activity and safety) [15,59,60] Invasion/Scaffolding proteins: FAK PROTAC: Simultaneously eliminates kinase and scaffolding functions [19] HDAC8, PTK6, HSP90α degraders [61,62,63] Apoptosis regulation: MDM2 PROTAC: Active in p53-mutant TNBC via TAp73 activation [64]	Core contradiction: Potent degradation activity vs. narrow therapeutic window [15]; CDK9 degrader B5 to P4 case demonstrates prodrug strategy can broaden window [60] Engineering necessity: Delivery systems, prodrug strategies, local administration are critical for translation Combination therapy: Synergistic potential with chemotherapy, PARPi, immunotherapy [65] Biomarkers: Urgent need to develop stratification systems based on target dependency, E3 expression, transcriptional signatures [66]

**Table 2:** Comparison of Major E3 Ligase Systems in BC PROTAC Research.

E3 Ligase (Complex)	Representative Recruiting Ligand	Mechanism and Design Considerations (in BC Context)	BC-Relevant Application Examples	Key Translational Considerations and Challenges
SCF^β-TRCP	IκBα phosphopeptide	Peptide-based PROTAC prototype validating “ternary complex-driven degradation.” Poor membrane permeability; now primarily a mechanistic research tool	ERα (proof-of-concept): Induced ERα degradation in cell-free systems, providing early evidence for the PROTAC concept [91]	Poor druggability of peptides (permeability, stability) Limited direct translational potential
CRL4^CRBN	Thalidomide and derivatives	IMiD-mediated “substrate reprogramming.” Most clinically advanced and widely applied E3 system	ER+: ARV-471 (Phase III, benefit in ESR1-mutant subgroup) [25]; AC699 (Phase I) [26] HER2+: Ab-PROTAC payload (BRD4 degradation) [36] TNBC: BET degradation (ARV-825) [49]; Liposomal CDK9 degrader [92]	Off-target risk: IMiD-induced neosubstrate degradation (IKZF1/3, etc.) [82,83] Acquired resistance: CRBN downregulation/mutation [34] Broad CRBN expression limits tumor selectivity
CRL2^VHL	VH032, VH298	Mimics HIF-1α hydroxyproline motif Often achieves subnanomolar potent degradation Strong ternary complex cooperativity [66,78]	ER+: ERD-308/148 (deep degradation of WT and mutant ERα) [93,94] HER2+: PI3K-p110α degradation (reverses lapatinib resistance) [16]; Ab-PROTAC payload (RIPK2 degradation) [95] TNBC: MDM2 degradation (YX-02-030) [64]; FAK [19], PTK6 [62] degradation; BRD4 degradation (synergy with cisplatin) [96]	Druggability challenges: beyond Rule of Five (bRo5) properties, low oral bioavailability, limited tumor penetration [32] Therapeutic window: Requires assessment of VHL tissue expression [88] Combination strategies need validation (potential antagonism with paclitaxel) [96]
cIAP1	Bestatin derivatives	SNIPER strategy: Simultaneous degradation of E3 and target Complex mechanism, may involve pleiotropic effects (e.g., ROS elevation)	ER+ (early all-small-molecule): SNIPER(ER) series achieved ERα degradation; some triggered ROS-dependent cell death [97,98,99]	E3 self-degradation may affect cellular homeostasis Narrow therapeutic window; requires fine-tuned concentration optimization
MDM2	Nutlin-3, etc.	Early validation of all-small-molecule PROTAC feasibility Now more commonly used as a degradation target rather than a recruiter	Platform validation: First all-small-molecule PROTAC (AR degradation) [100] As target: MDM2 degrader YX-02-030 active in p53-mutant TNBC [64]	Strong p53 pathway linkage: Therapeutic window assessment must consider p53 status Rarely used as a recruiter in modern designs
DCAF16	KB02 (covalent ligand)	Covalent recruitment strategy expands E3 toolkit Provides an alternative to circumvent CRBN/VHL dependence and resistance	TNBC: PROTAC A4 degrades CDK4/6 with preclinical antitumor activity [56]	Off-target covalent modification risk, immunogenicity High CMC complexity Expression profile and function in BC require elucidation [88]
UBR family	N-degron peptides (e.g., RLAA)	Harnesses N-end rule degradation pathway Offers an approach for proteins lacking small-molecule ligands (e.g., transcriptional regulators)	TNBC (proof-of-concept): SRC-1 degradation inhibits cancer cell migration and invasion [101]	Peptide stability and permeability remain major bottlenecks In vivo data lacking; far from translation

**Table 3:** Molecular Design Evolution of ER-Targeting PROTACs (Preclinical to Clinical).

Development Stage & Strategy	Representative Molecule/Strategy	Key Design Features & Tools	Key Validation Results & Scientific Significance
Proof-of-Concept (Peptide-Based/Hybrid)	IκBα phosphopeptide-estradiol conjugate	Phosphorylated peptide (recruits SCF^β-TRCP) + estradiol	First demonstration in cell-free systems that PROTACs can induce ERα ubiquitination and proteasome-dependent degradation; observed classic “Hook effect”; established ternary complex-driven degradation paradigm [91]
	Peptide-based VHL-recruiting PROTAC	VHL recognition peptide + estradiol	First intracellular ERα degradation achieved; systematically revealed critical impact of linker attachment site and length (C-7α optimal) on degradation activity, providing key principles for rational design [109]
Early All-Small-Molecule Exploration	SNIPER(ER) and SNIPER(ER)-3	Bestatin derivative (recruits cIAP1) + estrone/analogs	Validated feasibility of “all-small-molecule” PROTACs independent of peptides. SNIPER(ER)-3 triggered ROS-dependent cell death beyond degradation, suggesting degraders may produce complex cell-fate outputs distinct from pure antagonism [97,98]
Potent Degrader Era (Mainstream E3s)	ERD-308	VH032 (recruits VHL) + optimized ER ligand	Achieved subnanomolar degradation potency (DC_50_ ≈ 0.17 nM); degradation depth and antiproliferative activity superior to fulvestrant in preclinical models; established potency benchmark [94]
	ERD-148	VH032 (recruits VHL) + optimized ER ligand	Effectively degraded fulvestrant-insensitive phosphorylated ERα and ESR1 mutants; it mechanistically supports the concept that “deeper clearance can cover certain SERD-resistant scenarios” [93].
	Vepdegestrant	Pomalidomide derivative (recruits CRBN) + optimized ER ligand	First orally bioavailable ER PROTAC to enter the clinic. Preclinical: >95% degradation of WT and mutant ERα; superior antitumor efficacy vs. fulvestrant in multiple PDX models, with enhanced advantage in ESR1 Y537S mutant tumors [31]
Clinical Validation (Early-Stage)	AC699	CRBN-recruiting oral ERα PROTAC (structure undisclosed)	Phase I study (NCT05654532) in pretreated ER+/HER2− advanced BC: Overall ORR 33%, ESR1-mutant subgroup ORR 67%; supports clinical validation of ESR1 mutation as enrichment biomarker [26] (2024 ASCO Abstract)
Next-Generation Preclinical Candidates	UM-ERD-3111/UM-ERD-4001	Novel ER ligand scaffold; optimized linker exit vector to enhance oral exposure (E3 ligand undisclosed)	Reported ERα degradation potency (DC_50_ < 1 nM) and oral efficacy superior to ARV-471 in PDX models; achieved tumor regressions (2024 AACR Abstract)
	HP568	Fine-tuned ternary complex cooperativity to achieve mutant-selective degradation (E3 ligand undisclosed)	Maintained nanomolar degradation activity against multiple ESR1 mutants, including Y537S (DC_50_ 0.07−4 nM); synergistic antiproliferative effects with palbociclib (2024 AACR Abstract)
Mechanistic Expansion Strategies	ERE-PROTAC	Nucleic acid sequence (targets ER DBD) + E3 ligand	Targets DNA-binding domain (DBD) instead of LBD; provides proof-of-concept strategy to bypass LBD mutation-driven resistance [27]
	SRC-3 PROTAC	CRBN ligand + SRC-3 ligand SI-2	Targets key ER coactivator SRC-3; effective in ER+ and ESR1-mutant resistant models; suggests “coactivator degradation” as a complementary strategy to direct ER degradation [28]
	ERα/ARO Dual-Targeting PROTAC	Bifunctional molecule simultaneously targeting ERα and aromatase	Achieves combined clearance of “receptor signaling + ligand supply”; retains activity against ER-mutant cells in vitro and in vivo; potential value in suppressing resistance [29]

**Table 4:** PROTAC Research Strategies in HER2-Positive BC.

Strategy Category	Target/Pathway	Representative Molecule/Construct	Key Findings	References
Direct HER2 Degradation	HER2	Lapatinib-VHL PROTAC (Compound 1)	Induced EGFR and HER2 degradation in SKBr3 cells; antiproliferative activity superior to non-degrading control (IC_50_: 102 nM vs. 171 nM) Compared to inhibition, degradation resulted in more sustained downstream signal blockade (ERK, AKT) and attenuated kinome reprogramming	[35]
Antibody-PROTAC Conjugate	Payload: BRD4 Navigation: HER2	Trastuzumab-Ab-PROTAC3	HER2-mediated endocytosis enabled BRD4 degradation selectively in HER2-positive cells No degradation effect observed in HER2-negative cells Validated cascade mechanism: “HER2 binding → endocytosis → lysosomal trafficking → payload release → target degradation”	[36]
Antibody-PROTAC Conjugate	Payload: RIPK2 Navigation: HER2	Trastuzumab-PROTAC conjugate	Achieved selective RIPK2 degradation in HER2-positive cell lines Non-HER2-binding control antibody conjugate showed no degradation effect Demonstrated the target generality of this delivery strategy	[95]
Antibody-Modified Nanoparticle Delivery	Payload: BRD4 degrader MZ1 Navigation: HER2	MZ1-ACNPs (Trastuzumab-modified nanoparticles)	Nanoparticle system enhanced MZ1 uptake and cytotoxicity in HER2-positive cells Retained efficacy in models with intrinsic resistance to free MZ1 Suggested delivery systems can improve tumor accumulation and efficacy of PROTACs	[37]
Aptamer-PROTAC	Surface nucleolin (NCL)	AS1411 aptamer-PROTAC (ZL216)	Utilized AS1411 aptamer to target NCL overexpressed on the tumor cell surface Induced NCL degradation in vitro and in vivo; inhibited BC cell proliferation and migration	[38]
Degradation of Downstream Nodes	PI3K-p110α	p110α-selective PROTAC	Effectively degraded p110α in lapatinib-resistant cells and patient-derived organoids Resensitized resistant cells to lapatinib, with superior efficacy compared to the inhibitor alpelisib Suggested degradation of persistently activated downstream nodes as a strategy to overcome HER2-targeted therapy resistance	[16]
Degradation of Transcriptional Regulatory Nodes	CDK9	THAL-SNS-032	Induced CDK9 degradation in ER+/HER2+ cells and HER2-targeted therapy-resistant models; antiproliferative activity superior to parent inhibitor However, due to broad CDK9 expression, caused severe gastrointestinal toxicity in vivo, resulting in an extremely narrow therapeutic window	[15]

**Table 5:** PROTAC Target Landscape in TNBC.

Target	Functional Class	Representative PROTAC/Strategy	Key Mechanisms and Findings	References
BRD4	Transcriptional/Epigenetic Regulation	BETd-246, ARV-825, MZ1	Degradation superior to inhibition: Degradation more consistently downregulates proliferation/survival gene networks compared to inhibition, and overcomes BET inhibitor resistance in certain models Combination strategies: Synergy observed with cisplatin, but antagonism observed with paclitaxel, suggesting combinations require empirical validation Delivery optimization: Nanodelivery enhances tumor accumulation and may modulate the immune microenvironment (e.g., PD-L1 downregulation)	[49,50,65,96]
c-Myc	Transcriptional/Epigenetic Regulation	TNA-DNA bivalent binder-PROTAC-like strategy	Targeting “undruggable” proteins: Utilizes TNA aptamer and E-box DNA sequence as recognition elements to successfully degrade endogenous c-Myc/Max complex Combination synergy: Enhanced tumor growth inhibition when combined with palbociclib in TNBC models	[52]
PRMT5	Transcriptional/Epigenetic Regulation	CRBN-recruiting PRMT5 degrader	Epigenetic node degradation: Effectively degrades PRMT5 and inhibits tumor growth in TNBC cell lines, patient-derived organoids, and xenograft models Mechanistic linkage: Antitumor effects may involve downregulation of key transcription factors such as KLF5	[51]
PARP1	DNA Damage Repair (DDR)	NN3 and niraparib-based PROTACs	Novel mechanisms beyond inhibition: PARP1 degradation induces stronger apoptosis in TNBC cells with lower cytotoxicity to non-malignant cells Overcoming resistance: Triggers ferroptosis in p53-positive cancer cells via SLC7A11 downregulation; retains activity against PARP1 variants harboring resistance-associated mutations	[53,134,135]
CDK12/13	DNA Damage Repair/Transcriptional Regulation	BSJ-4-116, PP-C8, dual-targeting degraders	Transcription-coupled DDR node: Selective CDK12/13 degradation disrupts DDR gene transcription (e.g., premature cleavage and polyadenylation) Synthetic lethality synergy: Combination with PARP inhibitors produces synergistic antitumor effects	[54,55,136]
CDK4/6	Cell Cycle Regulation	Palbociclib-based PROTACs (CRBN/VHL/DCAF16-recruiting)	Deep cell cycle arrest: Achieves more thorough Rb pathway inhibition through protein elimination E3 diversification: Utilizing non-CRBN E3s (VHL, DCAF16) circumvents IMiD-related risks and provides alternatives following CRBN resistance	[56,57,58]
CDK9	Transcriptional Regulation	THAL-SNS-032 and prodrug P4	“Reverse therapeutic index” risk: Potent degrader exhibits an extremely narrow therapeutic window in vivo due to severe gastrointestinal toxicity Engineering solution: Conversion to TME-activated prodrug P4 successfully balances efficacy and safety, highlighting the necessity of prodrug strategies for highly toxic degraders	[15,59,60]
FAK	Invasion/Metastasis and Scaffolding Function	PROTAC-3	Eliminating scaffolding functions: FAK degradation suppresses TNBC cell migration and invasion more effectively than kinase inhibition alone Comprehensive advantage: Demonstrates the value of PROTACs in targeting proteins with both catalytic and non-catalytic functions	[19]
HDAC8	Invasion/Metastasis/Epigenetic Regulation	CT-4	Migration phenotype target: HDAC8 degradation inhibits cell migration significantly more potently than enzymatic inhibition; near-complete protein depletion markedly enhances anti-migratory activity	[61]
PTK6 (BRK)	Invasion/Metastasis and Scaffolding Function	VHL-recruiting PTK6 degrader	Covering non-catalytic functions: PTK6 degradation triggers stronger apoptosis and antiproliferative effects compared to inhibition alone, illustrating the advantage of the degradation strategy for multifunctional proteins	[62]
HSP90α	Chaperone/Invasion Metastasis	X10g (selective HSP90α degrader)	Isoform selectivity to reduce toxicity: Selective degradation of HSP90α isoform demonstrates superior antitumor efficacy and reduced systemic toxicity compared to pan-HSP90 inhibitor BP3 in preclinical models	[63,145]
G9a/GLP	Epigenetic Regulation/Invasion Metastasis	G9a/GLP PROTAC	Selective migration inhibition: G9a/GLP degradation significantly suppresses BC cell migration without strongly affecting proliferation, suggesting potential as a metastasis-specific intervention node	[146]
MDM2	Tumor Suppression and Apoptosis Regulation	YX-02-030, natural product-derived PROTACs	p53-independent apoptosis reactivation: In p53-mutant TNBC, MDM2 degradation reactivates apoptotic programs through alternative pathways, such as activation of p53 family member TAp73 Expanding therapeutic scope: Provides a novel strategy for MDM2 targeting in p53-mutant contexts	[64,147]

## Data Availability

Data sharing does not apply to this article as no new datasets were generated or analyzed during the current study. All discussed information is based on previously published literature.

## Abbreviations

Abbreviation	Full term
Ab–PROTAC	Antibody–PROTAC conjugate
AbTAC	Antibody-based targeted chimera
ADC(s)	Antibody–drug conjugate(s)
AI(s)	Aromatase inhibitor(s)
AKT	Protein kinase B
AR	Androgen receptor
BC	Breast cancer
BET	Bromodomain and extra-terminal (family)
BICR	Blinded independent central review
BRD4	Bromodomain-containing protein 4
CMC	Chemistry, manufacturing, and controls
CRBN	Cereblon
ctDNA	Circulating tumor DNA
DC_50_	Half-maximal degradation concentration
DDR	DNA damage response
DLT(s)	Dose-limiting toxicity(ies)
Dmax	Maximum degradation
E1	Ubiquitin-activating enzyme
E2	Ubiquitin-conjugating enzyme
E3	E3 ubiquitin ligase
EGFR	Epidermal growth factor receptor
ER	Estrogen receptor
ERα	Estrogen receptor alpha
ERK	Extracellular signal-regulated kinase
ESR1	Estrogen receptor 1
FAK	Focal adhesion kinase
GSH	Glutathione
HDAC8	Histone deacetylase 8
HER2	Human epidermal growth factor receptor 2
HIF-1α	Hypoxia-inducible factor-1 alpha
HR	Hazard ratio
HRD	Homologous recombination deficiency
HSP90α	Heat shock protein 90 alpha
IAP	Inhibitor of apoptosis protein
IC_50_	Half-maximal inhibitory concentration
IMiD	Immunomodulatory imide drug
ITT	Intent-to-treat
LBD	Ligand-binding domain
LYTAC(s)	Lysosome-targeting chimera(s)
MDM2	Mouse double minute 2 homolog
MMP-2	Matrix metalloproteinase-2
mTOR	Mechanistic target of rapamycin
ORR	Objective response rate
OS	Overall survival
PARP1	Poly(ADP-ribose) polymerase 1
PD	Pharmacodynamics
PD-L1	Programmed death-ligand 1
PDT	Photodynamic therapy
PDX(s)	Patient-derived xenograft(s)
PFS	Progression-free survival
P-gp	P-glycoprotein
PI3K	Phosphoinositide 3-kinase
PK	Pharmacokinetics
POI	Protein of interest
PR	Progesterone receptor
PROTAC(s)	Proteolysis-targeting chimera(s)
Rb	Retinoblastoma protein
RCT(s)	Randomized controlled trial(s)
Ro5	Rule of Five
ROS	Reactive oxygen species
RTK	Receptor tyrosine kinase
SERD(s)	Selective estrogen receptor degrader(s)
SRC	Steroid receptor coactivator
TME	Tumor microenvironment
TNBC	Triple-negative breast cancer
TP53	Tumor protein p53
TPD	Targeted protein degradation
TRAIL	Tumor necrosis factor-related apoptosis-inducing ligand
Ub	Ubiquitin
UPS	Ubiquitin–proteasome system
VHL	Von Hippel–Lindau

## References

[1] Bray F., Laversanne M., Sung H., Ferlay J., Siegel R.L., Soerjomataram I., Jemal A. (2024). Global cancer statistics 2022: GLOBOCAN estimates of incidence and mortality worldwide for 36 cancers in 185 countries. CA Cancer J. Clin..

[2] Cardoso F., Spence D., Mertz S., Corneliussen-James D., Sabelko K., Gralow J., Cardoso M.J., Peccatori F., Paonessa D., Benares A. (2018). Global analysis of advanced/metastatic breast cancer: Decade report (2005–2015). Breast.

[3] Momenimovahed Z., Salehiniya H. (2019). Epidemiological characteristics of and risk factors for breast cancer in the world. Breast Cancer.

[4] Derakhshan F., Reis-Filho J.S. (2022). Pathogenesis of Triple-Negative Breast Cancer. Annu. Rev. Pathol..

[5] Xing P., Yang C., Hu H., Qian T., Xie B., Huang J., Wang Z. (2025). Antibody-drug conjugates in breast cancer: Current resistance mechanisms and future combination strategies. Cancer Drug Resist..

[6] Cameron D., Piccart-Gebhart M.J., Gelber R.D., Procter M., Goldhirsch A., de Azambuja E., Castro G., Untch M., Smith I., Gianni L. (2017). 11 years’ follow-up of trastuzumab after adjuvant chemotherapy in HER2-positive early breast cancer: Final analysis of the HERceptin Adjuvant (HERA) trial. Lancet.

[7] Wu Y., Sun R., Ren S., Zengin G., Li M. (2025). Neuronal Reshaping of the Tumor Microenvironment in Tumorigenesis and Metastasis: Bench to Clinic. Med. Adv..

[8] Song S., Liu Z., Wang Y., Gong B. (2025). Human Organoids and Their Application in Tumor Models, Disease Modeling, and Tissue Engineering. Med. Bull..

[9] Li X., Song Y. (2020). Proteolysis-targeting chimera (PROTAC) for targeted protein degradation and cancer therapy. J. Hematol. Oncol..

[10] Cardoso F., Senkus E., Costa A., Papadopoulos E., Aapro M., André F., Harbeck N., Aguilar Lopez B., Barrios C.H., Bergh J. (2018). 4th ESO-ESMO International Consensus Guidelines for Advanced Breast Cancer (ABC 4). Ann. Oncol..

[11] Rasha F., Sharma M., Pruitt K. (2021). Mechanisms of endocrine therapy resistance in breast cancer. Mol. Cell. Endocrinol..

[12] Jeselsohn R., Buchwalter G., De Angelis C., Brown M., Schiff R. (2015). ESR1 mutations—A mechanism for acquired endocrine resistance in breast cancer. Nat. Rev. Clin. Oncol..

[13] Toy W., Shen Y., Won H., Green B., Sakr R.A., Will M., Li Z., Gala K., Fanning S., King T.A. (2013). ESR1 ligand-binding domain mutations in hormone-resistant breast cancer. Nat. Genet..

[14] Razavi P., Chang M.T., Xu G., Bandlamudi C., Ross D.S., Vasan N., Cai Y., Bielski C.M., Donoghue M.T.A., Jonsson P. (2018). The Genomic Landscape of Endocrine-Resistant Advanced Breast Cancers. Cancer Cell.

[15] Noblejas-López M.D.M., Gandullo-Sánchez L., Galán-Moya E.M., López-Rosa R., Tébar-García D., Nieto-Jiménez C., Gómez-Juárez M., Burgos M., Pandiella A., Ocaña A. (2022). Antitumoral Activity of a CDK9 PROTAC Compound in HER2-Positive Breast Cancer. Int. J. Mol. Sci..

[16] Zhang H., Zhang L., He Y., Jiang D., Sun J., Luo Q., Liang H., Wang T., Li F., Tang Y. (2024). PI3K PROTAC overcomes the lapatinib resistance in PIK3CA-mutant HER2 positive breast cancer. Cancer Lett..

[17] Chen L., Zhou H., Wu H., Lu Q., Huang J., Wang S. (2024). Effect of immunotherapy or anti-angiogenesis therapy combined with chemotherapy for advanced triple-negative breast cancer: A real-world retrospective study. Int. Immunopharmacol..

[18] Gu S., Cui D., Chen X., Xiong X., Zhao Y. (2018). PROTACs: An Emerging Targeting Technique for Protein Degradation in Drug Discovery. Bioessays.

[19] Cromm P.M., Samarasinghe K.T.G., Hines J., Crews C.M. (2018). Addressing Kinase-Independent Functions of Fak via PROTAC-Mediated Degradation. J. Am. Chem. Soc..

[20] Alabi S.B., Crews C.M. (2021). Major advances in targeted protein degradation: PROTACs, LYTACs, and MADTACs. J. Biol. Chem..

[21] Martín-Acosta P., Xiao X. (2021). PROTACs to address the challenges facing small molecule inhibitors. Eur. J. Med. Chem..

[22] Bhatia N., Hazra S., Thareja S. (2023). Selective Estrogen receptor degraders (SERDs) for the treatment of breast cancer: An overview. Eur. J. Med. Chem..

[23] Churcher I. (2018). Protac-Induced Protein Degradation in Drug Discovery: Breaking the Rules or Just Making New Ones?. J. Med. Chem..

[24] Chamberlain P.P., Hamann L.G. (2019). Development of targeted protein degradation therapeutics. Nat. Chem. Biol..

[25] Campone M., De Laurentiis M., Jhaveri K., Hu X., Ladoire S., Patsouris A., Zamagni C., Cui J., Cazzaniga M., Cil T. (2025). Vepdegestrant, a PROTAC Estrogen Receptor Degrader, in Advanced Breast Cancer. N. Engl. J. Med..

[26] Patel M.R. (2024). Preliminary results from a phase 1 study of AC699, an orally bioavailable chimeric estrogen receptor degrader, in patients with advanced or metastatic breast cancer. J. Clin. Oncol..

[27] Zhang X., Zhang Z., Xue X., Fan T., Tan C., Liu F., Tan Y., Jiang Y. (2022). PROTAC Degrader of Estrogen Receptor α Targeting DNA-Binding Domain in Breast Cancer. ACS Pharmacol. Transl. Sci..

[28] Liang J., Wang D., Wu Y., Shi J., Xie B., Xiao R., Ni J., Wang C., Dong C., Shu H.B. (2025). Intercepting the Downstream of the Estrogen Receptor Signaling Pathway: Discovery of a Potent and Efficient SRC-3 PROTAC Degrader for Overcoming Endocrine Resistance Breast Cancer. J. Med. Chem..

[29] Xin L., Wang C., Cheng Y., Wang H., Guo X., Deng X., Deng X., Xie B., Hu H., Min C. (2024). Discovery of Novel ERα and Aromatase Dual-Targeting PROTAC Degraders to Overcome Endocrine-Resistant Breast Cancer. J. Med. Chem..

[30] Bidard F.C., Hardy-Bessard A.C., Dalenc F., Bachelot T., Pierga J.Y., de la Motte Rouge T., Sabatier R., Dubot C., Frenel J.S., Ferrero J.M. (2022). Switch to fulvestrant and palbociclib versus no switch in advanced breast cancer with rising ESR1 mutation during aromatase inhibitor and palbociclib therapy (PADA-1): A randomised, open-label, multicentre, phase 3 trial. Lancet Oncol..

[31] Gough S.M., Flanagan J.J., Teh J., Andreoli M., Rousseau E., Pannone M., Bookbinder M., Willard R., Davenport K., Bortolon E. (2024). Oral Estrogen Receptor PROTAC Vepdegestrant (ARV-471) Is Highly Efficacious as Monotherapy and in Combination with CDK4/6 or PI3K/mTOR Pathway Inhibitors in Preclinical ER+ Breast Cancer Models. Clin. Cancer Res..

[32] Wu M., Zhao Y., Zhang C., Pu K. (2024). Advancing Proteolysis Targeting Chimera (PROTAC) Nanotechnology in Protein Homeostasis Reprograming for Disease Treatment. ACS Nano.

[33] Zhong J., Zhao R., Wang Y., Su Y.X., Lan X. (2024). Nano-PROTACs: State of the art and perspectives. Nanoscale.

[34] Zhang L., Riley-Gillis B., Vijay P., Shen Y. (2019). Acquired Resistance to BET-PROTACs (Proteolysis-Targeting Chimeras) Caused by Genomic Alterations in Core Components of E3 Ligase Complexes. Mol. Cancer Ther..

[35] Burslem G.M., Smith B.E., Lai A.C., Jaime-Figueroa S., McQuaid D.C., Bondeson D.P., Toure M., Dong H., Qian Y., Wang J. (2018). The Advantages of Targeted Protein Degradation Over Inhibition: An RTK Case Study. Cell Chem. Biol..

[36] Maneiro M.A., Forte N., Shchepinova M.M., Kounde C.S., Chudasama V., Baker J.R., Tate E.W. (2020). Antibody-PROTAC Conjugates Enable HER2-Dependent Targeted Protein Degradation of BRD4. ACS Chem. Biol..

[37] Cimas F.J., Niza E., Juan A., Noblejas-López M.D.M., Bravo I., Lara-Sanchez A., Alonso-Moreno C., Ocaña A. (2020). Controlled Delivery of BET-PROTACs: In Vitro Evaluation of MZ1-Loaded Polymeric Antibody Conjugated Nanoparticles in Breast Cancer. Pharmaceutics.

[38] Zhang L., Li L., Wang X., Liu H., Zhang Y., Xie T., Zhang H., Li X., Peng T., Sun X. (2022). Development of a novel PROTAC using the nucleic acid aptamer as a targeting ligand for tumor selective degradation of nucleolin. Mol. Ther. Nucleic Acids.

[39] Mrhalová M., Kodet R., Kalinová M., Hilská I. (2003). Relative quantification of ERBB2 mRNA in invasive duct carcinoma of the breast: Correlation with ERBB-2 protein expression and ERBB2 gene copy number. Pathol. Res. Pract..

[40] Krishnamurti U., Silverman J.F. (2014). HER2 in breast cancer: A review and update. Adv. Anat. Pathol..

[41] Sorkin A., Goh L.K. (2009). Endocytosis and intracellular trafficking of ErbBs. Exp. Cell Res..

[42] Bonifacino J.S., Traub L.M. (2003). Signals for sorting of transmembrane proteins to endosomes and lysosomes. Annu. Rev. Biochem..

[43] Haglund K., Dikic I. (2012). The role of ubiquitylation in receptor endocytosis and endosomal sorting. J. Cell Sci..

[44] Singareeka Raghavendra A., Damodaran S., Barcenas C.H., Fuqua S.A., Layman R.M., Tripathy D. (2026). Personalizing therapies over the course of hormone receptor-positive/HER2-negative metastatic breast cancer. CA Cancer J. Clin..

[45] Gao J., Hou B., Zhu Q., Yang L., Jiang X., Zou Z., Li X., Xu T., Zheng M., Chen Y.H. (2022). Engineered bioorthogonal POLY-PROTAC nanoparticles for tumour-specific protein degradation and precise cancer therapy. Nat. Commun..

[46] Perou C.M., Sørlie T., Eisen M.B., van de Rijn M., Jeffrey S.S., Rees C.A., Pollack J.R., Ross D.T., Johnsen H., Akslen L.A. (2000). Molecular portraits of human breast tumours. Nature.

[47] Garrido-Castro A.C., Lin N.U., Polyak K. (2019). Insights into Molecular Classifications of Triple-Negative Breast Cancer: Improving Patient Selection for Treatment. Cancer Discov..

[48] Li Y., Zhang H., Merkher Y., Chen L., Liu N., Leonov S., Chen Y. (2022). Recent advances in therapeutic strategies for triple-negative breast cancer. J. Hematol. Oncol..

[49] Noblejas-López M.D.M., Nieto-Jimenez C., Burgos M., Gómez-Juárez M., Montero J.C., Esparís-Ogando A., Pandiella A., Galán-Moya E.M., Ocaña A. (2019). Activity of BET-proteolysis targeting chimeric (PROTAC) compounds in triple negative breast cancer. J. Exp. Clin. Cancer Res..

[50] Bai L., Zhou B., Yang C.Y., Ji J., McEachern D., Przybranowski S., Jiang H., Hu J., Xu F., Zhao Y. (2017). Targeted Degradation of BET Proteins in Triple-Negative Breast Cancer. Cancer Res..

[51] Guo Y., Li Y., Zhou Z., Hou L., Liu W., Ren W., Mi D., Sun J., Dai X., Wu Y. (2024). Targeting PRMT5 through PROTAC for the treatment of triple-negative breast cancer. J. Exp. Clin. Cancer Res..

[52] Li X., Zhang Z., Gao F., Ma Y., Wei D., Lu Z., Chen S., Wang M., Wang Y., Xu K. (2023). c-Myc-Targeting PROTAC Based on a TNA-DNA Bivalent Binder for Combination Therapy of Triple-Negative Breast Cancer. J. Am. Chem. Soc..

[53] Ossovskaya V., Koo I.C., Kaldjian E.P., Alvares C., Sherman B.M. (2010). Upregulation of Poly (ADP-Ribose) Polymerase-1 (PARP1) in Triple-Negative Breast Cancer and Other Primary Human Tumor Types. Genes. Cancer.

[54] Niu T., Li K., Jiang L., Zhou Z., Hong J., Chen X., Dong X., He Q., Cao J., Yang B. (2022). Noncovalent CDK12/13 dual inhibitors-based PROTACs degrade CDK12-Cyclin K complex and induce synthetic lethality with PARP inhibitor. Eur. J. Med. Chem..

[55] Jiang B., Gao Y., Che J., Lu W., Kaltheuner I.H., Dries R., Kalocsay M., Berberich M.J., Jiang J., You I. (2021). Discovery and resistance mechanism of a selective CDK12 degrader. Nat. Chem. Biol..

[56] Pu C., Liu Y., Deng R., Xu Q., Wang S., Zhang H., Luo D., Ma X., Tong Y., Li R. (2023). Development of PROTAC degrader probe of CDK4/6 based on DCAF16. Bioorg. Chem..

[57] Steinebach C., Ng Y.L.D., Sosič I., Lee C.S., Chen S., Lindner S., Vu L.P., Bricelj A., Haschemi R., Monschke M. (2020). Systematic exploration of different E3 ubiquitin ligases: An approach towards potent and selective CDK6 degraders. Chem. Sci..

[58] Zhao B., Burgess K. (2019). PROTACs suppression of CDK4/6, crucial kinases for cell cycle regulation in cancer. Chem. Commun..

[59] Wei D., Wang H., Zeng Q., Wang W., Hao B., Feng X., Wang P., Song N., Kan W., Huang G. (2021). Discovery of Potent and Selective CDK9 Degraders for Targeting Transcription Regulation in Triple-Negative Breast Cancer. J. Med. Chem..

[60] Hu H., Wang Y., Wang M., Zhang Z., Gu X., Sun R., Liu X., Li N., Ding N., Li W. (2025). Translational Research on the Oral Delivery of the Cytotoxic PROTAC Molecule via Tumor-Targeting Prodrug Strategy for Triple-Negative Breast Cancer Treatment. J. Med. Chem..

[61] Zhao C., Chen D., Suo F., Setroikromo R., Quax W.J., Dekker F.J. (2023). Discovery of highly potent HDAC8 PROTACs with anti-tumor activity. Bioorg. Chem..

[62] Martinez C., Xiong Y., Bartkowski A., Harada I., Ren X., Byerly J., Port E., Jin J., Irie H. (2025). A PROTAC degrader suppresses oncogenic functions of PTK6, inducing apoptosis of breast cancer cells. Cell Chem. Biol..

[63] Jiang Q., Fu M., Tang Y., Li G., Tu G., Wu X., Wu Q., Huang X., Xu J., Liu Y. (2023). Discovery of X10g as a selective PROTAC degrader of Hsp90α protein for treating breast cancer. Eur. J. Med. Chem..

[64] Adams C.M., Mitra R., Xiao Y., Michener P., Palazzo J., Chao A., Gour J., Cassel J., Salvino J.M., Eischen C.M. (2023). Targeted MDM2 Degradation Reveals a New Vulnerability for p53-Inactivated Triple-Negative Breast Cancer. Cancer Discov..

[65] Chen X., Li F., Cui B., Yan Q., Qiu C., Zhu Z., Wen L., Chen W. (2025). Liposomes-mediated enhanced antitumor effect of docetaxel with BRD4-PROTAC as synergist for breast cancer chemotherapy/immunotherapy. Int. J. Pharm..

[66] Kumar H., Sobhia M.E. (2024). Interplay of PROTAC Complex Dynamics for Undruggable Targets: Insights into Ternary Complex Behavior and Linker Design. ACS Med. Chem. Lett..

[67] Amm I., Sommer T., Wolf D.H. (2014). Protein quality control and elimination of protein waste: The role of the ubiquitin-proteasome system. Biochim. Biophys. Acta.

[68] Pettersson M., Crews C.M. (2019). PROteolysis TArgeting Chimeras (PROTACs)—Past, present and future. Drug Discov. Today Technol..

[69] Kim H., Park J., Kim J.M. (2022). Targeted Protein Degradation to Overcome Resistance in Cancer Therapies: PROTAC and N-Degron Pathway. Biomedicines.

[70] Ocaña A., Pandiella A. (2020). Proteolysis targeting chimeras (PROTACs) in cancer therapy. J. Exp. Clin. Cancer Res..

[71] Gao H., Sun X., Rao Y. (2020). PROTAC Technology: Opportunities and Challenges. ACS Med. Chem. Lett..

[72] Cowan A.D., Ciulli A. (2022). Driving E3 Ligase Substrate Specificity for Targeted Protein Degradation: Lessons from Nature and the Laboratory. Annu. Rev. Biochem..

[73] Sakamoto K.M., Kim K.B., Kumagai A., Mercurio F., Crews C.M., Deshaies R.J. (2001). Protacs: Chimeric molecules that target proteins to the Skp1-Cullin-F box complex for ubiquitination and degradation. Proc. Natl. Acad. Sci. USA.

[74] Corson T.W., Aberle N., Crews C.M. (2008). Design and Applications of Bifunctional Small Molecules: Why Two Heads Are Better Than One. ACS Chem. Biol..

[75] Sosič I., Bricelj A., Steinebach C. (2022). E3 ligase ligand chemistries: From building blocks to protein degraders. Chem. Soc. Rev..

[76] Burslem G.M., Crews C.M. (2017). Small-Molecule Modulation of Protein Homeostasis. Chem. Rev..

[77] Radhakrishnan S., Hoff O., Muellner M.K. (2022). Current Challenges in Small Molecule Proximity-Inducing Compound Development for Targeted Protein Degradation Using the Ubiquitin Proteasomal System. Molecules.

[78] Gadd M.S., Testa A., Lucas X., Chan K.H., Chen W., Lamont D.J., Zengerle M., Ciulli A. (2017). Structural basis of PROTAC cooperative recognition for selective protein degradation. Nat. Chem. Biol..

[79] Dong Y., Ma T., Xu T., Feng Z., Li Y., Song L., Yao X., Ashby C.R., Hao G.F. (2024). Characteristic roadmap of linker governs the rational design of PROTACs. Acta Pharm. Sin. B.

[80] Cyrus K., Wehenkel M., Choi E.Y., Han H.J., Lee H., Swanson H., Kim K.B. (2011). Impact of linker length on the activity of PROTACs. Mol. BioSyst..

[81] Ito T., Ando H., Suzuki T., Ogura T., Hotta K., Imamura Y., Yamaguchi Y., Handa H. (2010). Identification of a primary target of thalidomide teratogenicity. Science.

[82] Lu G., Middleton R.E., Sun H., Naniong M., Ott C.J., Mitsiades C.S., Wong K.K., Bradner J.E., Kaelin W.G. (2014). The myeloma drug lenalidomide promotes the cereblon-dependent destruction of Ikaros proteins. Science.

[83] Krönke J., Udeshi N.D., Narla A., Grauman P., Hurst S.N., McConkey M., Svinkina T., Heckl D., Comer E., Li X. (2014). Lenalidomide causes selective degradation of IKZF1 and IKZF3 in multiple myeloma cells. Science.

[84] Furihata H., Yamanaka S., Honda T., Miyauchi Y., Asano A., Shibata N., Tanokura M., Sawasaki T., Miyakawa T. (2020). Structural bases of IMiD selectivity that emerges by 5-hydroxythalidomide. Nat. Commun..

[85] Min J.H., Yang H., Ivan M., Gertler F., Kaelin W.G., Pavletich N.P. (2002). Structure of an HIF-1alpha -pVHL complex: Hydroxyproline recognition in signaling. Science.

[86] Hon W.C., Wilson M.I., Harlos K., Claridge T.D., Schofield C.J., Pugh C.W., Maxwell P.H., Ratcliffe P.J., Stuart D.I., Jones E.Y. (2002). Structural basis for the recognition of hydroxyproline in HIF-1 alpha by pVHL. Nature.

[87] Itoh Y., Ishikawa M., Naito M., Hashimoto Y. (2010). Protein knockdown using methyl bestatin-ligand hybrid molecules: Design and synthesis of inducers of ubiquitination-mediated degradation of cellular retinoic acid-binding proteins. J. Am. Chem. Soc..

[88] Chen Y., Liu F., Pal S., Hu Q. (2024). Proteolysis-targeting drug delivery system (ProDDS): Integrating targeted protein degradation concepts into formulation design. Chem. Soc. Rev..

[89] Cyrus K., Wehenkel M., Choi E.Y., Lee H., Swanson H., Kim K.B. (2010). Jostling for position: Optimizing linker location in the design of estrogen receptor-targeting PROTACs. ChemMedChem.

[90] Frost A., O’Connor S., Ciulli A. (2026). Allosteric PROTACs: Expanding the Horizon of Targeted Protein Degradation. J. Am. Chem. Soc..

[91] Sakamoto K.M., Kim K.B., Verma R., Ransick A., Stein B., Crews C.M., Deshaies R.J. (2003). Development of Protacs to target cancer-promoting proteins for ubiquitination and degradation. Mol. Cell. Proteom..

[92] Arenas-Moreira M., Noblejas-López M.D.M., Ripoll C., Moya-López C., Díaz-Tejeiro C., Ocaña A., Martin-Ezama L., Bravo I., Alonso-Moreno C. (2025). Liposomal formulation of the CDK9 PROTAC THAL-SNS-032 enhances the antitumor activity in breast cancer cell lines. Biomed. Pharmacother..

[93] Hu B., Hu J. (2024). Complete elimination of estrogen receptor α by PROTAC estrogen receptor α degrader ERD-148 in breast cancer cells. Breast Cancer Res. Treat..

[94] Hu J., Hu B., Wang M., Xu F., Miao B., Yang C.Y., Wang M., Liu Z., Hayes D.F., Chinnaswamy K. (2019). Discovery of ERD-308 as a Highly Potent Proteolysis Targeting Chimera (PROTAC) Degrader of Estrogen Receptor (ER). J. Med. Chem..

[95] Chan K., Sathyamurthi P.S., Queisser M.A., Mullin M., Shrives H., Coe D.M., Burley G.A. (2023). Antibody-Proteolysis Targeting Chimera Conjugate Enables Selective Degradation of Receptor-Interacting Serine/Threonine-Protein Kinase 2 in HER2+ Cell Lines. Bioconjug. Chem..

[96] Hu J., Yang J., Zhou R., Chen K., Zhao H., Zhou Y. (2025). Discovery of a potent BRD4 PROTAC and evaluation of its bioactivity in breast cancer cell lines. Biochem. Pharmacol..

[97] Itoh Y., Kitaguchi R., Ishikawa M., Naito M., Hashimoto Y. (2011). Design, synthesis and biological evaluation of nuclear receptor-degradation inducers. Bioorg. Med. Chem..

[98] Okuhira K., Demizu Y., Hattori T., Ohoka N., Shibata N., Nishimaki-Mogami T., Okuda H., Kurihara M., Naito M. (2013). Development of hybrid small molecules that induce degradation of estrogen receptor-alpha and necrotic cell death in breast cancer cells. Cancer Sci..

[99] Okuhira K., Demizu Y., Hattori T., Ohoka N., Shibata N., Kurihara M., Naito M. (2016). Molecular Design, Synthesis, and Evaluation of SNIPER(ER) That Induces Proteasomal Degradation of ERα. Methods Mol. Biol..

[100] Schneekloth A.R., Pucheault M., Tae H.S., Crews C.M. (2008). Targeted intracellular protein degradation induced by a small molecule: En route to chemical proteomics. Bioorg. Med. Chem. Lett..

[101] Lee Y., Heo J., Jeong H., Hong K.T., Kwon D.H., Shin M.H., Oh M., Sable G.A., Ahn G.O., Lee J.S. (2020). Targeted Degradation of Transcription Coactivator SRC-1 through the N-Degron Pathway. Angew. Chem. Int. Ed. Engl..

[102] Chirnomas D., Hornberger K.R., Crews C.M. (2023). Protein degraders enter the clinic—A new approach to cancer therapy. Nat. Rev. Clin. Oncol..

[103] Bidard F.C., Kaklamani V.G., Neven P., Streich G., Montero A.J., Forget F., Mouret-Reynier M.A., Sohn J.H., Taylor D., Harnden K.K. (2022). Elacestrant (oral selective estrogen receptor degrader) Versus Standard Endocrine Therapy for Estrogen Receptor-Positive, Human Epidermal Growth Factor Receptor 2-Negative Advanced Breast Cancer: Results From the Randomized Phase III EMERALD Trial. J. Clin. Oncol..

[104] Arao Y., Korach K.S. (2021). The physiological role of estrogen receptor functional domains. Essays Biochem..

[105] McDonnell D.P., Wardell S.E., Chang C.Y., Norris J.D. (2021). Next-Generation Endocrine Therapies for Breast Cancer. J. Clin. Oncol..

[106] Hanzl A., Casement R., Imrichova H., Hughes S.J., Barone E., Testa A., Bauer S., Wright J., Brand M., Ciulli A. (2023). Functional E3 ligase hotspots and resistance mechanisms to small-molecule degraders. Nat. Chem. Biol..

[107] Fanning S.W., Mayne C.G., Dharmarajan V., Carlson K.E., Martin T.A., Novick S.J., Toy W., Green B., Panchamukhi S., Katzenellenbogen B.S. (2016). Estrogen receptor alpha somatic mutations Y537S and D538G confer breast cancer endocrine resistance by stabilizing the activating function-2 binding conformation. Elife.

[108] Neven P., Han S.N. (2025). PROTAC SERD vepdegestrant outperforms fulvestrant for advanced-stage ER(+)HER2(-) breast cancer harbouring acquired ESR1 mutations. Nat. Rev. Clin. Oncol..

[109] Rodriguez-Gonzalez A., Cyrus K., Salcius M., Kim K., Crews C.M., Deshaies R.J., Sakamoto K.M. (2008). Targeting steroid hormone receptors for ubiquitination and degradation in breast and prostate cancer. Oncogene.

[110] Cyrus K., Wehenkel M., Choi E.Y., Swanson H., Kim K.B. (2010). Two-headed PROTAC: An effective new tool for targeted protein degradation. ChemBioChem.

[111] Jiang Y., Deng Q., Zhao H., Xie M., Chen L., Yin F., Qin X., Zheng W., Zhao Y., Li Z. (2018). Development of Stabilized Peptide-Based PROTACs against Estrogen Receptor α. ACS Chem. Biol..

[112] Dai Y., Yue N., Gong J., Liu C., Li Q., Zhou J., Huang W., Qian H. (2020). Development of cell-permeable peptide-based PROTACs targeting estrogen receptor α. Eur. J. Med. Chem..

[113] Gonzalez T.L., Hancock M., Sun S., Gersch C.L., Larios J.M., David W., Hu J., Hayes D.F., Wang S., Rae J.M. (2020). Correction to: Targeted degradation of activating estrogen receptor α ligand-binding domain mutations in human breast cancer. Breast Cancer Res. Treat..

[114] Wolfson E., Goldenberg M., Solomon S., Frishberg A., Pinkas-Kramarski R. (2016). Nucleolin-binding by ErbB2 enhances tumorigenicity of ErbB2-positive breast cancer. Oncotarget.

[115] Zhao L., Han X., Lu J., McEachern D., Wang S. (2020). A highly potent PROTAC androgen receptor (AR) degrader ARD-61 effectively inhibits AR-positive breast cancer cell growth in vitro and tumor growth in vivo. Neoplasia.

[116] Paiva S.L., Crews C.M. (2019). Targeted protein degradation: Elements of PROTAC design. Curr. Opin. Chem. Biol..

[117] Banik S.M., Pedram K., Wisnovsky S., Ahn G., Riley N.M., Bertozzi C.R. (2020). Lysosome-targeting chimaeras for degradation of extracellular proteins. Nature.

[118] Zhang Y., Chang D., Zhang J. (2017). Research Advances in Resistance to Platinum-based Chemotherapy in Lung Cancer. Zhongguo Yi Xue Ke Xue Yuan Xue Bao.

[119] Hallett R.M., Dvorkin-Gheva A., Bane A., Hassell J.A. (2012). A gene signature for predicting outcome in patients with basal-like breast cancer. Sci. Rep..

[120] Horiuchi D., Kusdra L., Huskey N.E., Chandriani S., Lenburg M.E., Gonzalez-Angulo A.M., Creasman K.J., Bazarov A.V., Smyth J.W., Davis S.E. (2012). MYC pathway activation in triple-negative breast cancer is synthetic lethal with CDK inhibition. J. Exp. Med..

[121] Sahni J.M., Keri R.A. (2018). Targeting bromodomain and extraterminal proteins in breast cancer. Pharmacol. Res..

[122] Kong Y., Lan T., Wang L., Gong C., Lv W., Zhang H., Zhou C., Sun X., Liu W., Huang H. (2024). BRD4-specific PROTAC inhibits basal-like breast cancer partially through downregulating KLF5 expression. Oncogene.

[123] Lehmann B.D., Bauer J.A., Chen X., Sanders M.E., Chakravarthy A.B., Shyr Y., Pietenpol J.A. (2011). Identification of human triple-negative breast cancer subtypes and preclinical models for selection of targeted therapies. J. Clin. Investig..

[124] Gerratana L., Basile D., Buono G., De Placido S., Giuliano M., Minichillo S., Coinu A., Martorana F., De Santo I., Del Mastro L. (2018). Androgen receptor in triple negative breast cancer: A potential target for the targetless subtype. Cancer Treat. Rev..

[125] Gucalp A., Tolaney S., Isakoff S.J., Ingle J.N., Liu M.C., Carey L.A., Blackwell K., Rugo H., Nabell L., Forero A. (2013). Phase II trial of bicalutamide in patients with androgen receptor-positive, estrogen receptor-negative metastatic Breast Cancer. Clin. Cancer Res..

[126] Bonnefoi H., Grellety T., Tredan O., Saghatchian M., Dalenc F., Mailliez A., L’Haridon T., Cottu P., Abadie-Lacourtoisie S., You B. (2016). A phase II trial of abiraterone acetate plus prednisone in patients with triple-negative androgen receptor positive locally advanced or metastatic breast cancer (UCBG 12-1). Ann. Oncol..

[127] Traina T.A., Miller K., Yardley D.A., Eakle J., Schwartzberg L.S., O’Shaughnessy J., Gradishar W., Schmid P., Winer E., Kelly C. (2018). Enzalutamide for the Treatment of Androgen Receptor-Expressing Triple-Negative Breast Cancer. J. Clin. Oncol..

[128] Lehmann B.D., Abramson V.G., Sanders M.E., Mayer E.L., Haddad T.C., Nanda R., Van Poznak C., Storniolo A.M., Nangia J.R., Gonzalez-Ericsson P.I. (2020). TBCRC 032 IB/II Multicenter Study: Molecular Insights to AR Antagonist and PI3K Inhibitor Efficacy in Patients with AR(+) Metastatic Triple-Negative Breast Cancer. Clin. Cancer Res..

[129] Lim B., Seth S., Yam C., Huo L., Fujii T., Lee J., Bassett R., Nasser S., Ravenberg L., White J. (2024). Phase 2 study of neoadjuvant enzalutamide and paclitaxel for luminal androgen receptor-enriched TNBC: Trial results and insights into “ARness”. Cell Rep. Med..

[130] Bonnefoi H., Lerebours F., Pulido M., Arnedos M., Tredan O., Dalenc F., Guiu S., Teixeira L., Mollon D., Levy C. (2025). Darolutamide or capecitabine in triple-negative, androgen receptor-positive, advanced breast cancer (UCBG 3-06 START): A multicentre, non-comparative, randomised, phase 2 trial. Lancet Oncol..

[131] Jones P., Wilcoxen K., Rowley M., Toniatti C. (2015). Niraparib: A Poly(ADP-ribose) Polymerase (PARP) Inhibitor for the Treatment of Tumors with Defective Homologous Recombination. J. Med. Chem..

[132] Blazek D., Kohoutek J., Bartholomeeusen K., Johansen E., Hulinkova P., Luo Z., Cimermancic P., Ule J., Peterlin B.M. (2011). The Cyclin K/Cdk12 complex maintains genomic stability via regulation of expression of DNA damage response genes. Genes Dev..

[133] Tadesse S., Duckett D.R., Monastyrskyi A. (2021). The promise and current status of CDK12/13 inhibition for the treatment of cancer. Future Med. Chem..

[134] Zhao Q., Lan T., Su S., Rao Y. (2019). Induction of apoptosis in MDA-MB-231 breast cancer cells by a PARP1-targeting PROTAC small molecule. Chem. Commun..

[135] Li G., Lin S.S., Yu Z.L., Wu X.H., Liu J.W., Tu G.H., Liu Q.Y., Tang Y.L., Jiang Q.N., Xu J.H. (2022). A PARP1 PROTAC as a novel strategy against PARP inhibitor resistance via promotion of ferroptosis in p53-positive breast cancer. Biochem. Pharmacol..

[136] Yang J., Chang Y., Tien J.C., Wang Z., Zhou Y., Zhang P., Huang W., Vo J., Apel I.J., Wang C. (2022). Discovery of a Highly Potent and Selective Dual PROTAC Degrader of CDK12 and CDK13. J. Med. Chem..

[137] Wang H., Ba J., Kang Y., Gong Z., Liang T., Zhang Y., Qi J., Wang J. (2023). Recent Progress in CDK4/6 Inhibitors and PROTACs. Molecules.

[138] Kumarasamy V., Gao Z., Zhao B., Jiang B., Rubin S.M., Burgess K., Witkiewicz A.K., Knudsen E.S. (2023). PROTAC-mediated CDK degradation differentially impacts cancer cell cycles due to heterogeneity in kinase dependencies. Br. J. Cancer.

[139] Wu T., Qin Z., Tian Y., Wang J., Xu C., Li Z., Bian J. (2020). Recent Developments in the Biology and Medicinal Chemistry of CDK9 Inhibitors: An Update. J. Med. Chem..

[140] Sonawane Y.A., Taylor M.A., Napoleon J.V., Rana S., Contreras J.I., Natarajan A. (2016). Cyclin Dependent Kinase 9 Inhibitors for Cancer Therapy. J. Med. Chem..

[141] Sengupta S., Biarnes M.C., Jordan V.C. (2014). Cyclin dependent kinase-9 mediated transcriptional de-regulation of cMYC as a critical determinant of endocrine-therapy resistance in breast cancers. Breast Cancer Res. Treat..

[142] Lemke J., von Karstedt S., Abd El Hay M., Conti A., Arce F., Montinaro A., Papenfuss K., El-Bahrawy M.A., Walczak H. (2014). Selective CDK9 inhibition overcomes TRAIL resistance by concomitant suppression of cFlip and Mcl-1. Cell Death Differ..

[143] Gan X., Wang F., Luo J., Zhao Y., Wang Y., Yu C., Chen J. (2024). Proteolysis Targeting Chimeras (PROTACs) based on celastrol induce multiple protein degradation for triple-negative breast cancer treatment. Eur. J. Pharm. Sci..

[144] Gu Y., Yang M., Wang W., Li L., Ma Y., Liu W., Zhao Q. (2025). YX-112, a novel celastrol-derived PROTAC, inhibits the development of triple-negative breast cancer by targeting the degradation of multiple proteins. Front. Pharmacol..

[145] Liu Q., Tu G., Hu Y., Jiang Q., Liu J., Lin S., Yu Z., Li G., Wu X., Tang Y. (2022). Discovery of BP3 as an efficacious proteolysis targeting chimera (PROTAC) degrader of HSP90 for treating breast cancer. Eur. J. Med. Chem..

[146] Mukherjee A., Yamashita Y., Maeda R., Akiyama T., Endo K., Takada Y., Tsumoto H., Moriyama Y., Ito A., Shirai F. (2025). A Novel PROTAC G9a/GLP Degrader that Inhibits, Similar to G9a siRNA, the Migration of MCF-7 Breast-Cancer Cells without Affecting Proliferation. J. Med. Chem..

[147] Li Y., Li G., Zuo C., Wang X., Han F., Jia Y., Shang H., Tian Y. (2024). Discovery of ganoderic acid A (GAA) PROTACs as MDM2 protein degraders for the treatment of breast cancer. Eur. J. Med. Chem..

[148] Weiner T.M., Liu E.T., Craven R.J., Cance W.G. (1993). Expression of focal adhesion kinase gene and invasive cancer. Lancet.

[149] Shibue T., Brooks M.W., Inan M.F., Reinhardt F., Weinberg R.A. (2012). The outgrowth of micrometastases is enabled by the formation of filopodium-like protrusions. Cancer Discov..

[150] Shimizu T., Fukuoka K., Takeda M., Iwasa T., Yoshida T., Horobin J., Keegan M., Vaickus L., Chavan A., Padval M. (2016). A first-in-Asian phase 1 study to evaluate safety, pharmacokinetics and clinical activity of VS-6063, a focal adhesion kinase (FAK) inhibitor in Japanese patients with advanced solid tumors. Cancer Chemother. Pharmacol..

[151] Shah S.P., Roth A., Goya R., Oloumi A., Ha G., Zhao Y., Turashvili G., Ding J., Tse K., Haffari G. (2012). The clonal and mutational evolution spectrum of primary triple-negative breast cancers. Nature.

[152] Hamilton E. (2022). Abstract PD13-08: First-in-human safety and activity of ARV-471, a novel PROTAC® estrogen receptor degrader, in ER+/HER2- locally advanced or metastatic breast cancer. Cancer Res..

[153] Iwata H., Naito Y., Hattori M., Yoshimura A., Yonemori K., Aizawa M., Mori Y., Yoshimitsu J., Umeyama Y., Mukohara T. (2025). Safety and pharmacokinetics of vepdegestrant in Japanese patients with ER+ advanced breast cancer: A phase 1 study. Int. J. Clin. Oncol..

[154] Hamilton E.P. (2024). 218P Vepdegestrant, a proteolysis targeting chimera (PROTAC) estrogen receptor (ER) degrader, plus pal-bociclib (palbo) in ER+/human epidermal growth factor receptor 2 (HER2)- advanced breast cancer: Updated phase Ib cohort results. ESMO Open.

[155] Rej R.K. (2024). Abstract 4510: Potent and orally efficacious PROTAC degraders of estrogen receptor α (ERα). Cancer Res..

[156] Du W. (2024). Abstract LB179: Identification of the highly potent and orally available ER-targeting PROTAC degrader HP568 for the treatment of breast cancer. Cancer Res..

[157] Dragovich P.S., Adhikari P., Blake R.A., Blaquiere N., Chen J., Cheng Y.X., den Besten W., Han J., Hartman S.J., He J. (2020). Antibody-mediated delivery of chimeric protein degraders which target estrogen receptor alpha (ERα). Bioorg. Med. Chem. Lett..

[158] Elwyn G., Frosch D., Thomson R., Joseph-Williams N., Lloyd A., Kinnersley P., Cording E., Tomson D., Dodd C., Rollnick S. (2012). Shared decision making: A model for clinical practice. J. Gen. Intern. Med..

[159] Runowicz C.D., Leach C.R., Henry N.L., Henry K.S., Mackey H.T., Cowens-Alvarado R.L., Cannady R.S., Pratt-Chapman M.L., Edge S.B., Jacobs L.A. (2016). American Cancer Society/American Society of Clinical Oncology Breast Cancer Survivorship Care Guideline. CA Cancer J. Clin..

[160] Huang D., Zou Y., Huang H., Yin J., Long S., Sun W., Du J., Fan J., Chen X., Peng X. (2024). A PROTAC Augmenter for Photo-Driven Pyroptosis in Breast Cancer. Adv. Mater..

[161] Zhou Z., Fan H., Yu D., Shi F., Li Q., Zhang Z., Wang X., Zhang X., Dong C., Sun H. (2023). Glutathione-responsive PROTAC for targeted degradation of ERα in breast cancer cells. Bioorg. Med. Chem..

[162] Liu H., Ren C., Sun R., Wang H., Zhan Y., Yang X., Jiang B., Chen H. (2022). Reactive oxygen species-responsive Pre-PROTAC for tumor-specific protein degradation. Chem. Commun..

[163] Li Y., Zhao L., Li X.F. (2021). Targeting Hypoxia: Hypoxia-Activated Prodrugs in Cancer Therapy. Front. Oncol..

[164] Sharma N., Sarkar S., Ko T., Edwards K.J., Pham J.M., Nguyen T., Gong A.Z., Flores J., Trauner D., Sellmyer M.A. (2025). Photocontrolled trimethoprim PROTACs targeting the eDHFR protein tag. Nat. Commun..

[165] Yang C., Yang Y., Li Y., Ni Q., Li J. (2023). Radiotherapy-Triggered Proteolysis Targeting Chimera Prodrug Activation in Tumors. J. Am. Chem. Soc..

[166] Li Z., Jiang T., Yuan X., Li B., Wu C., Li Y., Huang Y., Xie X., Pan W., Ping Y. (2024). Controlled bioorthogonal activation of Bromodomain-containing protein 4 degrader by co-delivery of PROTAC and Pd-catalyst for tumor-specific therapy. J. Control. Release.

[167] Cheng X., Hu S., Cheng K. (2023). Microneedle Patch Delivery of PROTACs for Anti-Cancer Therapy. ACS Nano.

[168] Li H., Zhang Y., Shu H., Lv W., Su C., Nie F. (2022). Highlights in ultrasound-targeted microbubble destruction-mediated gene/drug delivery strategy for treatment of malignancies. Int. J. Pharm..

[169] Tong F., Wang Y., Xu Y., Zhou Y., He S., Du Y., Yang W., Lei T., Song Y., Gong T. (2024). MMP-2-triggered, mitochondria-targeted PROTAC-PDT therapy of breast cancer and brain metastases inhibition. Nat. Commun..

